# Access to highly specialized growth substrates and production of epithelial immunomodulatory metabolites determine survival of *Haemophilus influenzae* in human airway epithelial cells

**DOI:** 10.1371/journal.ppat.1010209

**Published:** 2022-01-27

**Authors:** Jennifer Hosmer, Marufa Nasreen, Rabeb Dhouib, Ama-Tawiah Essilfie, Horst Joachim Schirra, Anna Henningham, Emmanuelle Fantino, Peter Sly, Alastair G. McEwan, Ulrike Kappler

**Affiliations:** 1 School of Chemistry and Molecular Biosciences, Australian Infectious Diseases Research Centre, The University of Queensland, St. Lucia, Australia; 2 QIMR Berghofer Medical Research Institute, Herston, Australia; 3 Centre for Advanced Imaging, The University of Queensland, St. Lucia, Australia; 4 Child Health Research Centre, The University of Queensland, South Brisbane, Australia; University of Toronto, CANADA

## Abstract

*Haemophilus influenzae* (Hi) infections are associated with recurring acute exacerbations of chronic respiratory diseases in children and adults including otitis media, pneumonia, chronic obstructive pulmonary disease and asthma. Here, we show that persistence and recurrence of Hi infections are closely linked to Hi metabolic properties, where preferred growth substrates are aligned to the metabolome of human airway epithelial surfaces and include lactate, pentoses, and nucleosides, but not glucose that is typically used for studies of Hi growth *in vitro*. Enzymatic and physiological investigations revealed that utilization of lactate, the preferred Hi carbon source, required the LldD L-lactate dehydrogenase (conservation: 98.8% of strains), but not the two redox-balancing D-lactate dehydrogenases Dld and LdhA. Utilization of preferred substrates was directly linked to Hi infection and persistence. When unable to utilize L-lactate or forced to rely on salvaged guanine, Hi showed reduced extra- and intra-cellular persistence in a murine model of lung infection and in primary normal human nasal epithelia, with up to 3000-fold attenuation observed in competitive infections. In contrast, D-lactate dehydrogenase mutants only showed a very slight reduction compared to the wild-type strain. Interestingly, acetate, the major Hi metabolic end-product, had anti-inflammatory effects on cultured human tissue cells in the presence of live but not heat-killed Hi, suggesting that metabolic endproducts also influence HI-host interactions. Our work provides significant new insights into the critical role of metabolism for Hi persistence in contact with host cells and reveals for the first time the immunomodulatory potential of Hi metabolites.

## Author summary

The ability to access suitable growth substrates and use them for energy generation is essential for the survival of bacterial pathogens in their host but is often not well understood. This includes the highly prevalent respiratory pathogen *Haemophilus influenzae* (Hi), which causes pneumonia and otitis media, and worsens several common chronic respiratory infections such as asthma, bronchiectasis, and chronic obstructive pulmonary disease. Here we have shown that Hi metabolism is specifically adapted to the human respiratory tract, and access to preferred growth substrates, which included L-lactate, is required for Hi long-term persistence in primary human epithelia, including intracellular colonization of the host cells. The role of metabolism for host interactions was not limited to growth substrates but extended to Hi metabolic endproducts where we were able to link the immunometabolite acetate to a reduction of the host immune response to Hi infection. This is the first time that a specific link between metabolism and Hi persistence in the host has been established.

## Introduction

The success of *Haemophilus influenzae* (Hi) as a pathobiont is directly linked to its remarkable ability to persist in the human host and to access different epithelial niches. Hi asymptomatically colonizes the human nasopharynx with high adult carriage rates and initial infections occurring usually during the first two years of life [1,2]. Hi is involved in a variety of upper and lower respiratory tract diseases such as acute and recurrent otitis media, pneumonia and associated invasive diseases [3–8]. Additionally, Hi infection is an exacerbating factor for chronic diseases such as Chronic Obstructive Pulmonary Disease (COPD), Bronchiectasis, Cystic Fibrosis (CF) and Asthma, and has recently been identified as causing infectious sequelae in recovering COVID19 patients [4,8–10]. Current clinical isolates are typically non-typeable Hi strains (NTHi) that lack a polysaccharide capsule, and these are characterized by increasing antibiotic resistance and severity of the diseases they are associated with [11–13].

The ability of various bacterial pathogens to thrive intracellularly during infection of human tissues often correlates with increased persistence of infections and reduces the efficacy of antimicrobial therapy. Increasing evidence suggests that in addition to colonizing human epithelia extracellularly, Hi is able to enter into epithelial cells and survive intracellularly, including in resected respiratory tissues such as adenoids [14–16]. Following infection of human tissue cells, the presence of Hi both inside vacuolar compartments and as free bacteria in the tissue cell cytosol has been observed [17–20]. While uptake of NTHi into tissue cells has been linked to micropinocytosis and different host-cell receptors such as β-glucan receptor and platelet-activating factor (PAF) [19,21–23], there is currently no information about metabolic processes that enable Hi persistence in the cytosol of infected human cells. Existing studies indicate that Hi can grow on a variety of hexose sugars and possibly carboxylic acids *in vitro*, and individual metabolic enzymes such as pyruvate dehydrogenase and acetate kinase have been shown to play key roles in infection [24–27]. However, the links between Hi metabolic activities and colonization of specific cellular niches are only beginning to be understood.

Metabolic adaptation to various host cell environments has been studied in other human pathogens, both those found in the respiratory tract, such as *Mycobacterium tuberculosis* and *Legionella pneumophila* and those present in other body niches, such as *Salmonella enterica* and *Listeria monocytogenes* [28–31]. In these bacteria, changes in the preferentially used carbon sources have been associated with intra- and extracellular survival, but the carbon sources used by different bacterial species to access the host cytoplasm are diverse and include glucose, cholesterol, and branched-chain amino acids, indicating that these preferences may have evolved to suit the metabolic network of each pathogen [28,31].

Here, we have explored the nutritional preferences of five unrelated NTHi strains and investigated the role of preferred Hi growth substrates and key Hi metabolic endproducts for persistence and colonization. Our results demonstrate a clear association between the persistence of Hi infections and specific metabolic activities, including immunomodulatory effects of endproduct metabolites.

## Results

### *Hi* metabolism is highly adapted to carbon and nitrogen sources available during contact with human epithelia

**Fig 1:**
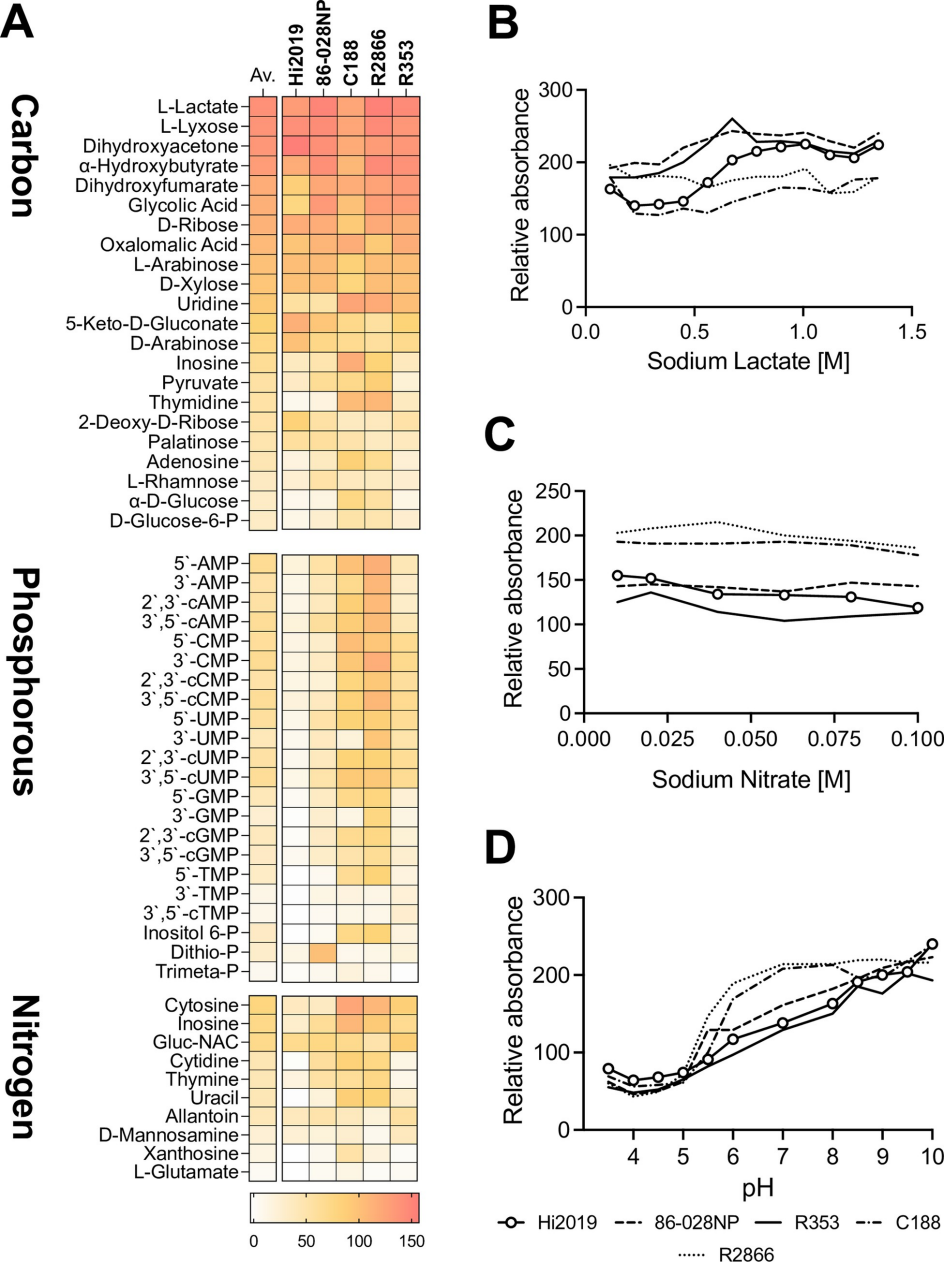
Metabolic and stress related properties of five, genetically unrelated NTHi strains. **A**: Carbon, Phosphorous, and Nitrogen sources used by NTHi strains (BIOLOG PM1,2,3,4). Both averages and individual absorbance scores obtained for each strain are shown (represented in a colour scale). Absorbance values are in arbitrary units as determined for each well by the Omnilog plate reader and relate to the colour intensity of the redox dye present in each well, where darker colour indicates stronger growth; **Panels B, C, D:** Growth of NTHi strains in the presence of osmolytes and different pH (BIOLOG PM 9,10). Values shown are omnilog absorbance readings taken after 24h of growth. **B**: Sodium lactate; **C**: Sodium nitrate; **D**: pH 3.5–10. NTHi strains used were Hi2019 (COPD; ST321), 86-028NP (otitis media; ST33), C188 (invasive; ST269), R2866 (invasive; ST99), Hi 535 (sputum, ST: unknown). For additional data, see S1 Fig and S1 Table.

The metabolic capabilities of five genetically diverse NTHi strains were mapped using Phenotypic Microarrays (Biolog, PM1-10 plates), uncovering a strong conservation of metabolic capabilities between strains, but also niche-specific substrate specialization. Only 34 out of 190 carbon sources tested were used by all strains, and only 13 carbon compounds supported strong growth in all strains (Fig 1A and S1 Table). These preferred carbon sources included several pentose sugars that are known components of human glycoproteins, but also endproducts and intermediates of cellular energy metabolism such as lactate, α-hydroxybutyrate, dihydroxyacetone and ribose (Fig 1A and S1 Table). Glucose, fructose and sialic acid that are commonly used for Hi *in vitro* growth were not part of this group of highly used carbon sources. The substrate specialization was even stronger for Hi nitrogen and phosphorous sources. Preferred N-sources for Hi were limited to purine and pyrimidine nucleosides and aminosugars such as N-Acetyl-D-Glucosamine and D-Mannosamine, while well-used P-sources were 3’- and 5’-nucleotide monophosphates, as well as cyclic nucleotide monophosphates and inositol hexa-phosphate (Fig 1A). This again suggests that glycoproteins and precursors of RNA and DNA components are major potential sources of Hi nutrients.

All five NTHi strains tested had similar physiological traits and were highly resistant to osmotic stressors such as formate, lactate, ammonium sulfate, nitrate and nitrite. NaCl and KCl were also well tolerated (Figs 1B, 1C, 1D and S1; data from PM plates 9 and 10 that test osmotic and pH stress; S1 Table). Optimal growth of NTHi was observed above pH 8.0, while below pH 5.5 growth was minimal. Two isolates from invasive disease patients (C188, R2866) showed some specific adaptations, including an increased ability to grow on glucose and nucleosides, and optimal growth at pH 7.0 and above, which is lower than that found for most other strains [32].

### Metabolomic analyses suggest that lactate utilization by NTHi occurs during co-culture with human epithelial cells

**Fig 2:**
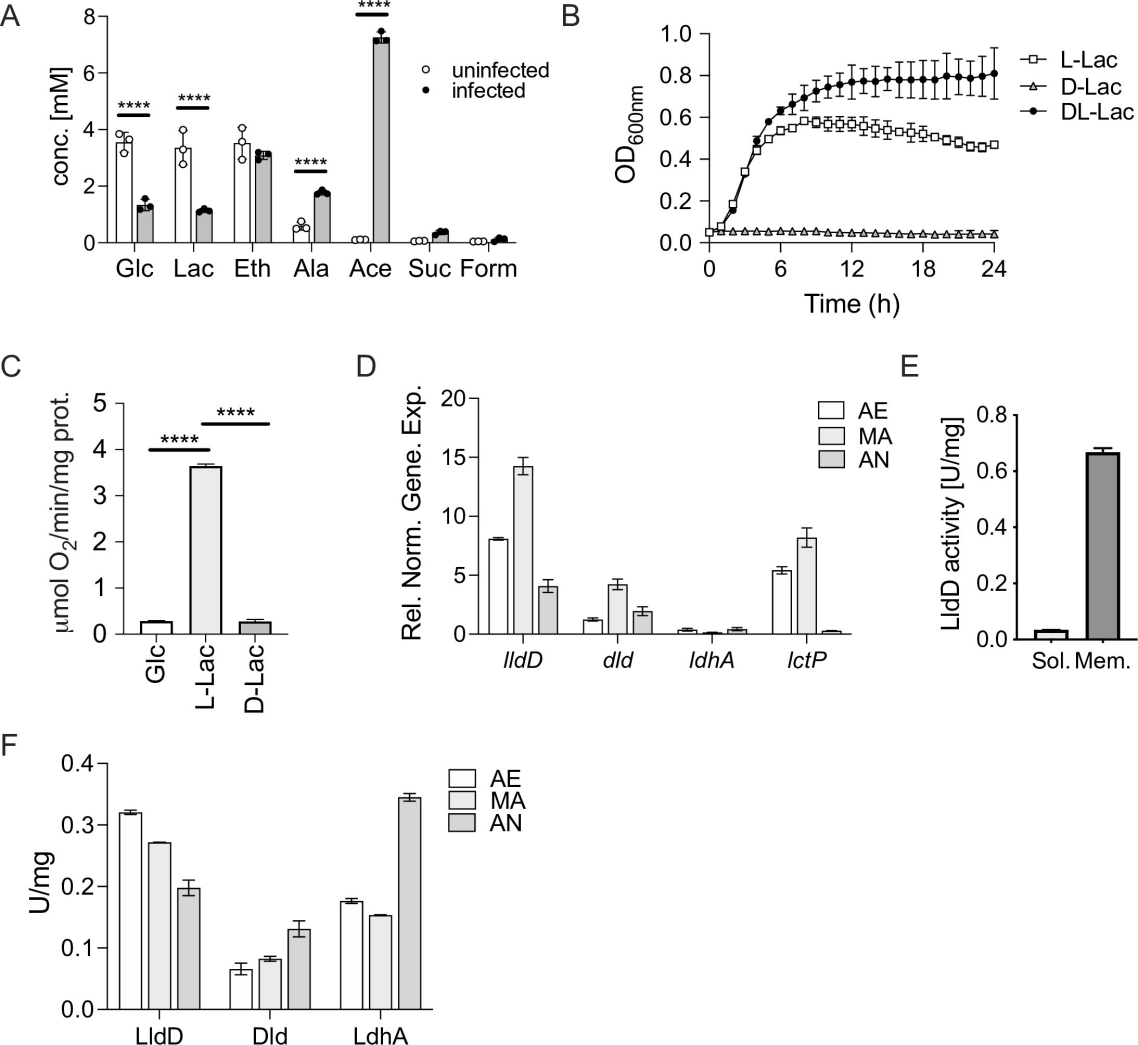
Lactate utilization by Hi2019^WT^. **Panels A, B:** Utilization of lactate by Hi2019^WT^ in tissue cell infections and *in vitro* growth assays **A:** ^1^H-NMR metabolomics of supernatants of 16HBE14 cultivated with or without Hi2019 infection (24h time point). **B:** Growth of Hi2019 on glucose (10mM), D- or L-lactate (25mM) as the primary carbon source in CDM medium under microaerobic (MA) conditions. **Panels C-F:** Activity and expression of lactate-converting enzymes in Hi2019^WT^ **C:** Oxygen-dependent respiration of Hi2019^WT^ whole cells with glucose (10mM), L- or D-lactate (10mM). **D:** Expression of Hi2019^WT^ lactate dehydrogenase (*lldD*, *dld*, *ldhA*) and lactate permease (*lctP*) genes under aerobic (AE), microaerobic (MA), and anaerobic (AN) conditions during exponential growth on D/L-lactate (25mM). Gene expression was normalized using *gyrA* as the reference gene. **E:** L-lactate dehydrogenase (LldD) activity in Hi2019^WT^ cell membranes and soluble cell extracts to determine enzyme location. **F:** Activities of Hi2019^WT^ lactate dehydrogenases, LldD (L-lactate), Dld (D-lactate), and LdhA (D-Lactate) under aerobic (AE), microaerobic (MA), and anaerobic (AN) conditions in cell extracts from cultures grown for 18 h with 25 mM D/L-lactate in CDM minimal medium. All data are shown as averages ± STDEV for 3 biological replicates; Statistical tests: Panel A: 2-way ANOVA with Sidak post-hoc test. Statistical significance is reported relative to the uninfected data for the same compound. Panel C: 1-way ANOVA, Tukey’s post-hoc test. Statistical significance is reported relative to respiration using L-lactate. **** = p<0.0001. Related data are shown in S2 Fig.

To determine the relevance of NTHi preferred carbon sources during epithelial cell infection, metabolite profiles of uninfected and NTHi-infected 16HBE14 bronchial epithelial tissue cells were determined by ^1^H-NMR, and this led to the identification of 32 metabolites (S2 Table). Uninfected 16HBE14 cells consumed the glucose present in MEM tissue culture medium as well as amino acids essential for human cell growth such as histidine, leucine, lysine and methionine. The 16HBE14 tissue cells produced lactate (3.35±0.60 mM), ethanol (3.52±0.56 mM) and alanine (0.59±0.14 mM), with alanine production probably resulting from the reaction of alanine transaminase [33] and the consumption of alanyl-glutamine present as the source of glutamine (Fig 2A and S2 Table). The production of lactate indicates that at least some of the glucose consumption is linked to glycolytic lactate production.

NTHi infection of 16HBE14 led to the production of known NTHi metabolic endproducts [34] such as acetate, succinate, and formate that were not present in the uninfected samples, and also resulted in lower final concentrations of glucose (1.33±0.20 mM) and lactate (1.13±0.06 mM) in the growth medium (Fig 2A). Alanine production increased (1.77±0.08 mM) compared to the uninfected tissue cell cultures, an observation that has previously been made following bacterial infection in rats and monkeys [35]. These data suggest that NTHi is likely driving the consumption of lactate and may have contributed to glucose utilization (Fig 2A). Additionally, increased glucose consumption may also be driven by the 16HBE14 cells, as increased glycolytic flux and resulting lactate production is a typical host cell reaction to infection [36]. However, we cannot exclude other possible explanations for the observed metabolite patterns such as a reduced production of lactate by the host cells, although based on the available literature this would appear to be less likely [36]. The 16HBE14 growth medium does not contain nucleobase derivatives, and significant amounts of nucleosides or nucleotides that are a major group of NTHi nutrients were not observed under these conditions (S2 Table).

### L-lactate supports strong NTHi growth and respiration

Our results indicated that lactate is a key carbon source for NTHi and we therefore explored the significance of lactate for NTHi growth and energy generation. On chemically defined medium (CDM) lacking all other major carbon sources, optimal growth of NTHi was achieved with 25 mM D/L- or L-lactate, while externally provided D-lactate did not support growth (Fig 2B). NTHi whole-cell respiratory activity with L-lactate as the substrate was 12.9- and 13.3-fold greater (p-value <0.0001) than for D-lactate or glucose, respectively (Fig 2C). This documents the importance of L-Lactate as a carbon source as well as a close link between L-lactate consumption and the NTHi respiratory chain. Growth on lactate as the primary carbon source led to a shift in the NTHi metabolic network, where acetate was the dominant metabolic product (S2A Fig), while under identical growth conditions with glucose as the carbon source acetate, succinate, and formate were produced.

### Hi lactate dehydrogenase activity varied in response to substrate availability and changes in the cellular redox environment

Analysis of Hi genomes revealed that most strains encode a lactate permease (LctP, WP_08034307.1) and three types of lactate dehydrogenases (LDH), a membrane-bound L-lactate dehydrogenase (LldD, EC1.1.5.B3) and two D-lactate dehydrogenases, the membrane-bound Dld (EC1.1.5.12) enzyme, and an NAD^+^-dependent LdhA (EC1.1.1.28). This combination of genes was found in 91.6% (598/653 strains) of NTHi strains examined, with another 48 strains (7.2%) containing *lldD*, *lctP* and *ldhA* genes but apparently lacking a *dld* gene (S4 Table). A small number of genetically divergent Hi strains (n = 5, 0.8%; symmetric identity: ~ 38%, gapped identity: ~ 82% to Hi2019 and Hi RdKW20) lacked two or more of the above proteins, and instead contained genes encoding a LutABC-type L-lactate utilization system and the LctP lactate permease [37], but no typical D-lactate dehydrogenases. This raises questions about the role of the two D-lactate dehydrogenases that are found in the majority of NTHi strains as D-lactate did not support NTHi growth, and fermentative D-lactate production was also not observed in NTHi growing on CDM [32,34].

In the COPD isolate strain Hi2019 [38] the genes encoding the lactate permease and the three lactate dehydrogenases were expressed during growth on glucose and growth on D/L-lactate, indicating that all four proteins are functionally relevant. The highest expression levels were observed for *lldD* and *lctP*, while *ldhA* was expressed at low levels throughout (Figs 2D and S2C).

Enzymatic activities of all three LDHs were also present in Hi2019, with higher activities following growth on lactate, suggesting that post-transcriptional regulation may be occurring, as gene expression did not vary significantly with carbon source (S2B and S2C Fig). However, variations in oxygen availability that NTHi can encounter during infection affected LDH activities. LldD activity decreased with increasing oxygen limitation, while the activities of Dld and LdhA increased under microaerobic and anaerobic conditions (Fig 2D). This suggests distinct roles for the NTHi D- and L-lactate dehydrogenases.

Kinetic parameters for the three Hi2019 LDHs were within the ranges reported for other enzymes of the same type [39–44] with *K*_M_app_ values of 0.30±0.03mM for LldD/L-lactate and 0.82±0.08mM for Dld/D-lactate with DCPIP as the electron acceptor (S5 Table). As described previously, the NAD^+^-dependent LdhA was essentially unidirectional and unable to oxidise D-lactate at an appreciable rate [45]. Dld and LldD activities in Hi2019 cell-free extracts (CFE) were strongly associated with the Hi cell membrane (LldD; 95%, Dld; 83%, S2D Fig), which was also observed in other bacteria and documents their connections to the Hi respiratory chain [46,47].

### Only the LldD L-lactate dehydrogenase is required for Hi growth on lactate-containing medium

To gain insights into the cellular roles of the NTHi lactate dehydrogenases, we constructed and verified deletion mutations in all three genes (S3 Fig). As expected, the Hi2019^Δ*lldD*^ strain was unable to grow on media containing D/L- or L-Lactate as the sole carbon source under all conditions tested, while growth of Hi2019^Δ*dld*^ and Hi2019^Δ*ldhA*^ strains was essentially unaffected (S3 Table). The growth of Hi2019^Δ*lldD*^ and Hi2019^Δ*dld*^ strains on glucose was reduced by ~ 30 and 60%, respectively, in the absence of oxygen, but comparable to the WT for aerobic and microaerobic conditions (S3 Table).

**Fig 3:**
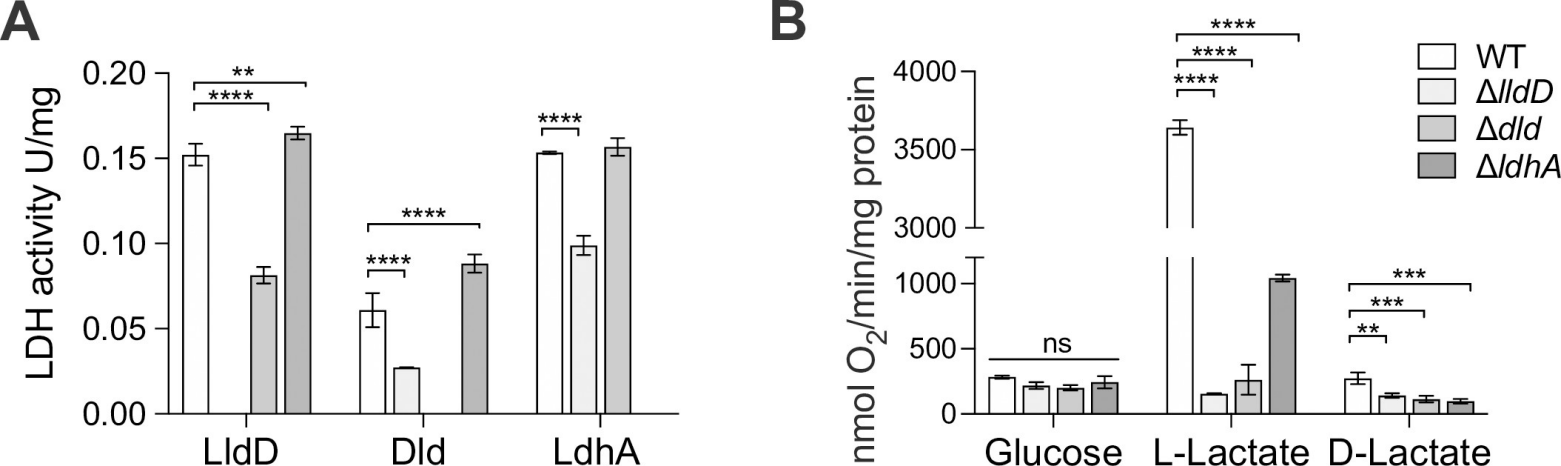
Hi lactate dehydrogenases are required for biomass production of biomass and redox balancing. **A:** Lactate dehydrogenase activities in Hi2019^WT^ and Hi2019 LDH mutant strains following growth on CDM medium with glucose (10 mM) **B:** Respiratory activities of Hi2019^WT^ and Hi2019 LDH mutant strains using glucose (10mM), L- or D-lactate (10mM) as substrates. All data are shown as averages ± STDEV for three independent assays; ** p<0.01, *** p<0.001, **** p<0.0001, 2-Way ANOVA with Dunnett’s post-hoc test (factors: bacterial strain & enzyme or respiratory substrate). Statistical significance is reported relative to the WT data. Related data are shown in S3 Fig.

In the Hi2019^Δ*lldD*^ strain, the relative activities of both Dld and LdhA were reduced, while LldD activity was reduced in Hi2019^Δ*dld*^. Loss of the soluble, NAD^+^-dependent LdhA did not affect activity of the other two lactate dehydrogenases (Fig 3A). Similarly, oxygen-dependent respiration with L-lactate was significantly reduced in both the Hi2019^Δ*lldD*^ and Hi2019^Δ*dld*^ strains (23-fold and 13-fold), but to a much lesser extent (3-fold) in Hi2019^Δ*ldhA*^ (Fig 3B). These data suggest that particularly the activities of the two membrane-bound LDH enzymes affect each other, possibly via changes to the cellular redox balance as both enzymes are connected to the Hi respiratory chain, and this might also cause the reduced growth of the respective mutant strains on glucose under anaerobic conditions.

### L-lactate dehydrogenase activity is required for NTHi survival in co-culture with human tissue cells and in a mouse model of lung infection

**Fig 4:**
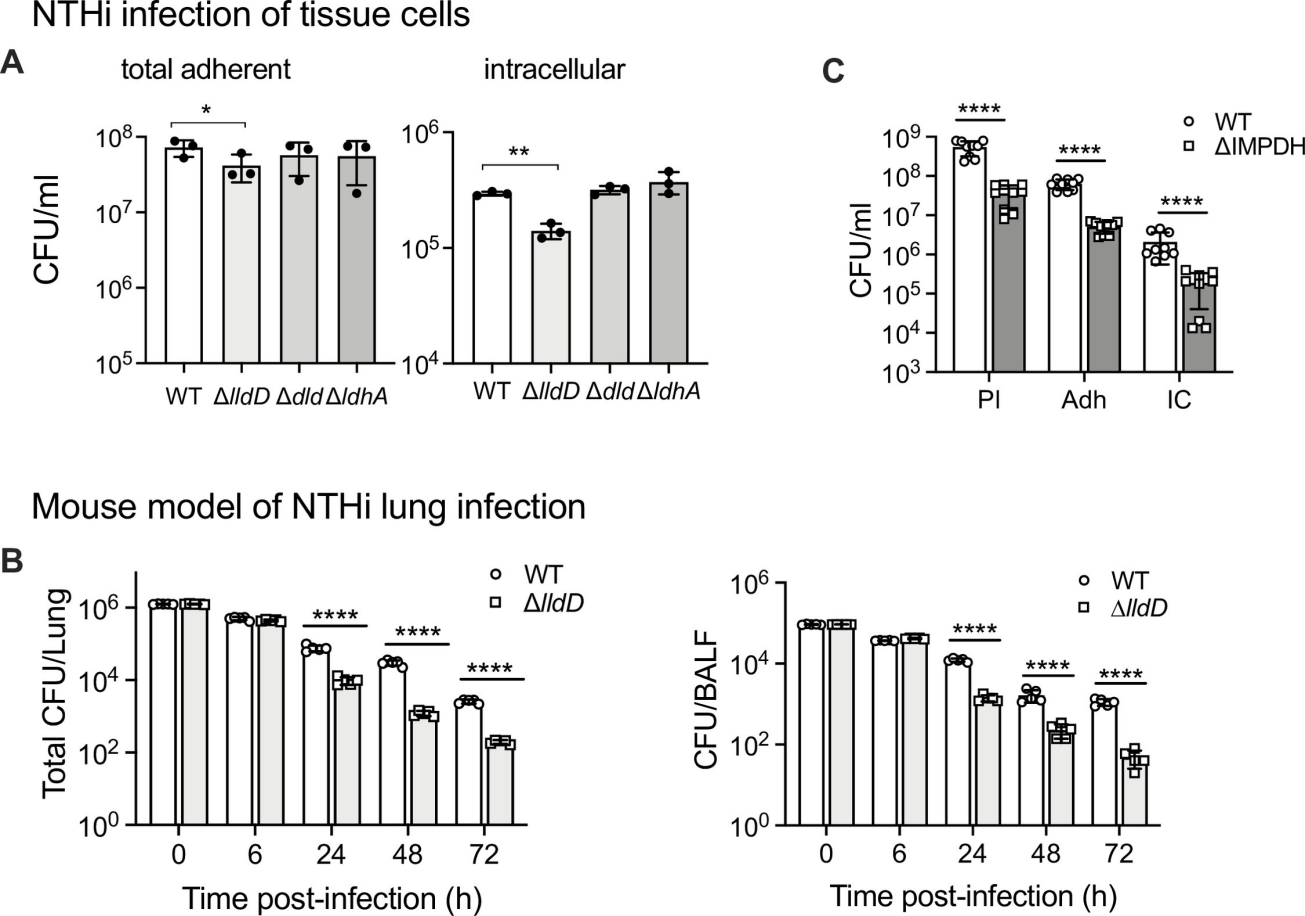
-lactate utilization and *de novo* purine synthesis are required for NTHi optimal fitness in tissue cell and mouse models of infection. L **A:** Infection of 16HBE14 bronchial epithelial cells with Hi2019^WT^ or Hi2019 LDH mutant strains. CFU/ml 24h post-infection are shown. Inoculum levels for all strains were ~ 1.5 x10^7^ CFU/ml. Assays used three biological replicates. **B:** Fitness of Hi2019^Δ*lldD*^ in a mouse model (Balb/c) of lung infection. CFU/lung and CFU present in bronchioalveolar lavage fluid (BALF) are shown. Inoculum levels for all mice and strains were ~ 1.1 x10^5^ CFU/ml, group size–n = 5 mice **C:** Infection of 16HBE14 bronchial epithelial cells with Hi2019^WT^ and the Hi2019 ImpDH mutant strain. Pl = planktonic, Ad = total adherent bacteria, IC = intracellular bacteria; 3 biological replicates were used per strain and assays, assays were repeated three times. All data are shown as averages of biological replicates ± STDEV; * p<0.05, ** p<0.01, *** p<0.001, **** p<0.0001; statistical significance is reported relative to the WT data for the same timepoint. Panels A: One-way ANOVA (Dunnett’s post-hoc test), Panel B & C: two-tailed, unpaired t-test, FDR-corrected. Related data are shown in S4 Fig.

We hypothesized that access to preferred growth substrates is required for NTHi fitness during infection, and we tested this for lactate using two commonly used models of NTHi infection, cultured tissue cells and a mouse model of lung infection. During infection of 16HBE14 bronchial epithelial tissue cells, the two D-lactate dehydrogenase mutant strains, Hi2019^*ΔldhA*^ and Hi2019^Δ*dld*^ behaved essentially like Hi2019^WT^ (Fig 4A). In contrast, for the Hi2019^Δ*lldD*^ strain, adherence to tissue cells was reduced 1.7-fold (p = 0.0120, 1-Way ANOVA) and invasion was reduced 2.1-fold (p = 0.0073, 1-Way ANOVA) 24 h post-infection. Interestingly, the excess glucose present in the MEM tissue culture medium was unable to rescue the host cell interaction defect in Hi2019^Δ*lldD*^, despite the fact that this strain can grow well on glucose-containing minimal media *in vitro* (Fig 4A and S3 Table).

Similar results were obtained for Hi2019^Δ*lldD*^ in a mouse model of lung infection. In this model, mice are inoculated intranasally with 10^7^ CFU, and as NTHi are unable to establish a stable infection in healthy mice, remaining NTHi CFU are determined at different time-points up to 72 h post-infection. While 6 h post-infection there was no reduction in bacterial loads in lung tissue and BAL fluid (BALF), 24 h, 48 h and 72 h post-infection Hi2019^Δ*lldD*^ CFUs were reduced by between 8- and 24-fold compared to the wildtype in both types of samples (Fig 4B). Giemsa staining of mouse immune cells in BALF revealed that, compared to Hi2019^WT^, influx of neutrophils was reduced during infection with Hi2019^Δ*lldD*^ while macrophage numbers showed statistically significant increases throughout (S4 Fig). Expression of genes encoding key cytokines (IL-6, TNFα, IL-1β) and BIRC3, which is involved in innate immune signalling and has been shown to increase in expression during infections of human cells with NTHi [48], were similar in mice infected with Hi2019^Δ*lldD*^ and Hi2019^WT^ at 6 h post-infection, while at 24 h and 48 h post-infection gene expression levels were reduced between 2.5- and 11-fold (S5 Fig, 2-Way ANOVA, p<0.05) in mice infected with Hi2019^Δ*lldD*^, in keeping with the reduction of Hi2019^Δ*lldD*^ CFUs recovered at these timepoints. Despite this difference in cytokine gene expression, levels of TNFα & IL-1β in BALF were generally similar for the Hi2019^WT^ and Hi2019^Δ*lldD*^ strain except for IL-1β levels at 6 h post-infection where lower levels were observed for infections with the mutant strain.

### Absence of *de novo* purine biosynthesis reduces NTHi fitness during infection

The Phenotypic Microarray data (Fig 1) indicated that nucleosides and nucleotides are an important source of carbon as well as nitrogen and phosphorous for Hi strains, and their uptake might be required during co-culture of Hi with host cells. To test this, we created a strain with a mutation in the *guaB* gene that encodes IMP dehydrogenase (ImpDH). ImpDH converts inosine monophosphate (IMP), the end product of the *de novo* purine biosynthesis pathway to xanthosine monophosphate, a precursor for the production of guanosine monophosphate (GMP). While a mutation of *guaB* should not affect the ability of NTHi to produce adenosine/adenine, it would prevent the formation of guanine as well as the use of inosine, which is highly used by NTHi as a carbon source and is a possible precursor of guanine. The ImpDH mutant strain would thus have to rely on purine salvage to obtain guanine for cell growth. In keeping with this, the Hi2019^Δ*ImpDH*^ strain was able to grow on the BHI complex medium, but growth on CDM that contains inosine as an additional carbon source required guanine supplementation. Interestingly, the Hi2019^Δ*ImpDH*^ strain was able to infect 16HBE14 tissue cells in the absence of guanine supplementation, even though cell numbers were reduced for planktonic (27-fold), total adherent (10-fold), and intracellular bacteria (12-fold) compared to Hi2019^WT^ (Fig 4C). These data show that the ImpDH mutation effectively abolishes *de novo* biosynthesis of guanine and also inosine utilization. However, the infection assay indicates that NTHi can effectively scavenge sufficient guanine for survival from the host cell environment, especially during attached and intracellular growth. Despite this, guanine scavenging from the host cells was insufficient to restore the wild-type phenotype, suggesting a significant role for guanine *de novo* synthesis during infection.

### Lactate utilization and purine biosynthesis are required for persistence of NTHi infections in primary human epithelia

As indicated above, mouse models only allow NTHi infections of limited duration, and to gain more detailed insights into the persistence of Hi infections we tested virulence of the Hi2019 wildtype and LDH mutant strains using differentiated normal human nasal epithelia (NHNE). NHNE are pseudostratified epithelia differentiated at Air-Liquid Interface (ALI) from primary human nasal cells and are a highly realistic model of human airway epithelia, the only known natural niche of NTHi [49,50]. The NHNE organoid model replicates the different types of epithelial cell populations, but does not contain resident immune cells. We infected the differentiated, air-exposed epithelial surface of NHNE with either Hi2019^WT^, one of the three LDH mutant strains or the IMPDH mutant strain at an MOI of 10:1. Total CFU, internalized CFU and CFU in the basal medium, indicative of migration of NTHi through the epithelium, were monitored for 7 days post-infection.

**Fig 5:**
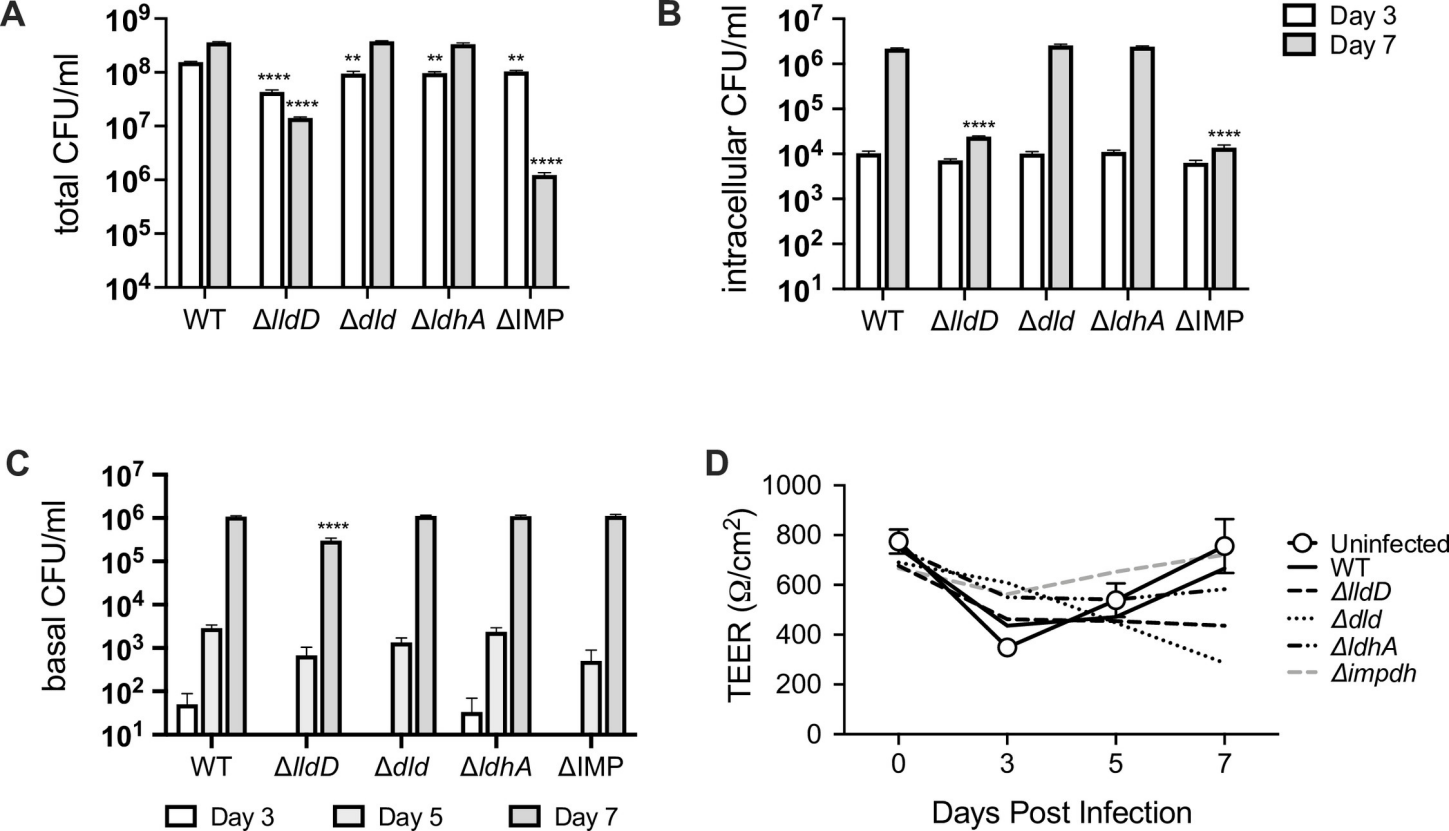
Infection of normal human nasal epithelia (NHNE) differentiated at the air-liquid interface (ALI) with Hi2019^WT^, lactate dehydrogenase, or the IMPDH mutant strains. **A:** Total bacterial loads (CFU/ml) at day 3 and day 7 post-infection **B:** Intracellular bacterial populations (CFU/ml) at day 3 and day 7 post-infection **C:** Bacteria (CFU/ml) present in the basal medium reservoir at day 3, day 5 and day 7 post-infection **D:** Transepithelial resistance (TEER) readings for infected and uninfected NHNE with similar readings reported across all NHNE indicating no damage from infection. Inoculum levels for all strains were ~ 3.8 x10^7^ CFU/ml. Two biological replicates were used per strain and time point, all data are shown as averages ± STDEV; ** p<0.01, **** p<0.0001, Statistical testing used 2-Way ANOVA (Variables: Bacterial strains; Time post-infection) and Dunnett’s post-hoc test. Statistical significance is reported relative to the WT data for the same timepoint and shown above the data point that is being compared to the WT value for clarity.

As had been observed for infection of cultured tissue cells, Hi2019^Δ*dld*^ and Hi2019^Δ*ldhA*^ behaved essentially like Hi2019^WT^ during NHNE infection. For these three strains total cell numbers reached ~ 3.5 x 10^8^ CFU/ml after 7 days (15–20 x increase above inoculum level), and ~ 2.3 x 10^6^ CFU/ml for intracellular bacteria (Fig 5A and 5B). In contrast, total cell numbers for Hi2019^Δ*lldD*^ maximally increased 2-fold above inoculum levels (day 3), with 1.42 x 10^7^ CFU/ml (70% of inoculum level) present 7 days after infection. Hi2019^Δ*lldD*^ also showed a progressively increasing attenuation in intracellular bacterial loads, where we observed a ~ 2-order of magnitude reduction (90-fold) compared to Hi2019^WT^ at day 7 (Fig 5B) documenting the importance of L-lactate utilization for Hi fitness during infection, and especially intracellular survival. For the Hi2019^Δ*ImpDH*^ strain intra- and extracellular survival were reduced 1.5-fold three days post-infection, however, by day 7 Hi2019^Δ*ImpDH*^ loads were reduced by over 2 orders of magnitude in both niches (159- and 291-times), revealing that guanine auxotrophy has a very strong impact on Hi persistence (Fig 5A and 5B). Interestingly, despite the generally high CFU-loads, NTHi infection did not significantly affect epithelial integrity as indicated by the similarity of transepithelial resistance readings (TEER) between infected and uninfected NHNE (Fig 5D).

Transepithelial migration of NTHi has been documented in clinical specimens, and here we used the presence of NTHi CFU/ml in the basal medium that provides nutrients to NHNE as an indicator of effective migration across the NHNE epithelia. Both Hi2019^WT^ and Hi2019^Δ*ldhA*^ were detectable in the basal medium in low numbers 3 days post-infection, while the Hi2019^Δ*dld*^, Hi2019^Δ*lldD*^ and Hi2019^Δ*ImpDH*^ strains were only detected 5 days post-infection. CFU counts remained low throughout for Hi2019^Δ*lldD*^ (3.6-4-fold reduction relative to Hi2019^WT^), while for all other strains CFU/ml were comparable to Hi2019^WT^ by day 7 (Fig 5C). Our results demonstrate that NTHi can migrate across human epithelia without causing major structural disruption, and that guanine availability in the basal medium was sufficient to support Hi2019^Δ*ImpDH*^ growth (Fig 5C and 5D).

### During competitive infections of NHNE Hi2019^WT^ outcompetes the *lldD* and *guaA* gene knockout strains

**Fig 6:**
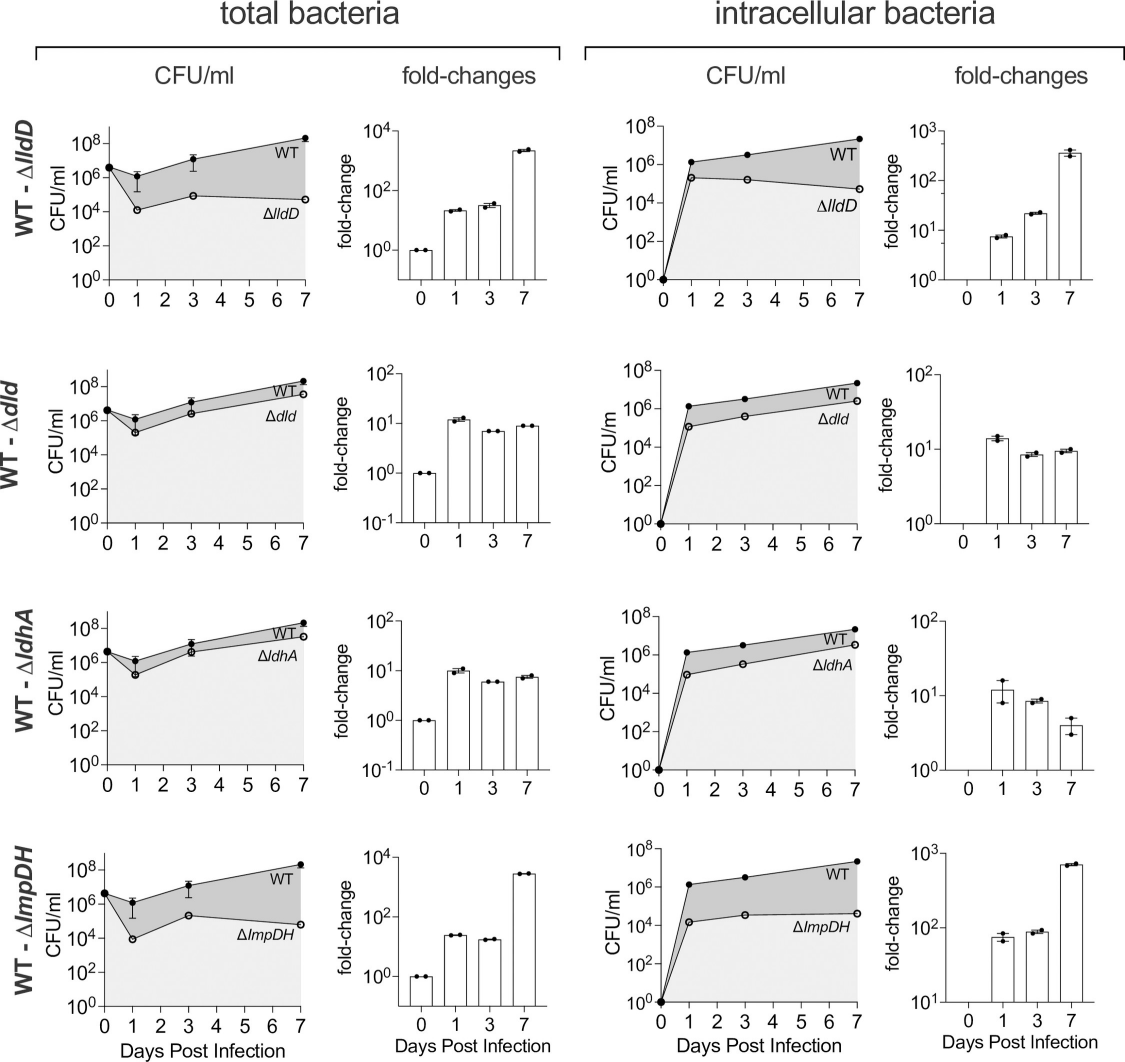
Competitive infection of NHNE with Hi2019^WT^ and Hi2019 LDH and the ImpDH mutant strains. NHNE (2 biological replicates per time point and strain combination) were infected using an inoculum that contained a 1:1 ratio of the Hi2019^WT^ & mutant strain. **Left:** Total bacterial loads **Right**: Intracellular bacterial loads. For each competition experiment, bacterial cell numbers (CFU/ml) at days 0–7 post-infection (line graph: WT: dark grey shading; mutant: light grey shading) and fold-changes (WT: mutant) are shown.

To better understand the reduced fitness/virulence of the *lldD* and *guaA/*ImpDH mutations, we used competitive infections of NHNE with equal amounts of Hi2019^WT^ and either the *lldD* or *ImpDH* mutant. Total and intracellular CFU/ml were monitored for seven days. The co-infection experiment confirmed the reduced virulence/persistence of the Hi2019^*ΔlldD*^ and Hi2019^*ΔImpDH*^ strains, in fact, for both strains phenotypes were more pronounced than in the single-strain infections, and for all strains we observed a reduction of total CFU on day 1 post-infection (Fig 6). Interestingly, in the competitive infection, the attenuation in total CFU/ml for the two mutant strains exceeded the attenuation of intracellular bacterial populations. For Hi2019^*ΔlldD*^ total CFU/ml were reduced by 23-, 27- and 2404-fold on days 1, 3 and 7 while intracellular CFU/ml were reduced by 7.5-, 21- and 361-fold at the respective time points (Fig 6). For Hi2019^*ΔImpDH*^ changes of 24.5-, 17.5- and 2783-fold and 66-, 88.5- and 705-fold were obtained for total and intracellular CFU/ml on days 1, 3, and 7, respectively (Fig 6). For both the Hi2019^*Δdld*^ and Hi2019^*ΔldhA*^ mutant strains that showed no phenotype in single infections, a consistent reduction (10-14-fold) in both total and intracellular cell numbers was obtained on day 1, while at later timepoints this difference became less pronounced. This documents that a small fitness defect, particularly during early colonization, is associated with a loss of the two D-lactate dehydrogenases. In contrast, for Hi2019^*ΔlldD*^ and Hi2019^*ΔImpDH*^ there was not only a significant reduction of CFU on day 1 post-infection, but both strains also failed to thrive between day 3 and day 7, revealing a role for the respective mutations in both early events of infection and long-term colonization (Fig 6).

Together, our data clearly show that L-lactate, identified as a key growth substrate using the phenotypic microarray system and guanine biosynthesis are directly relevant to Hi survival in contact with host cells and are key determinants for both extra- and intracellular survival.

### Attenuation of Lactate dehydrogenase and IMP dehydrogenase mutant strains could be affected by an underlying increase in sensitivity to oxidative stress

**Fig 7:**
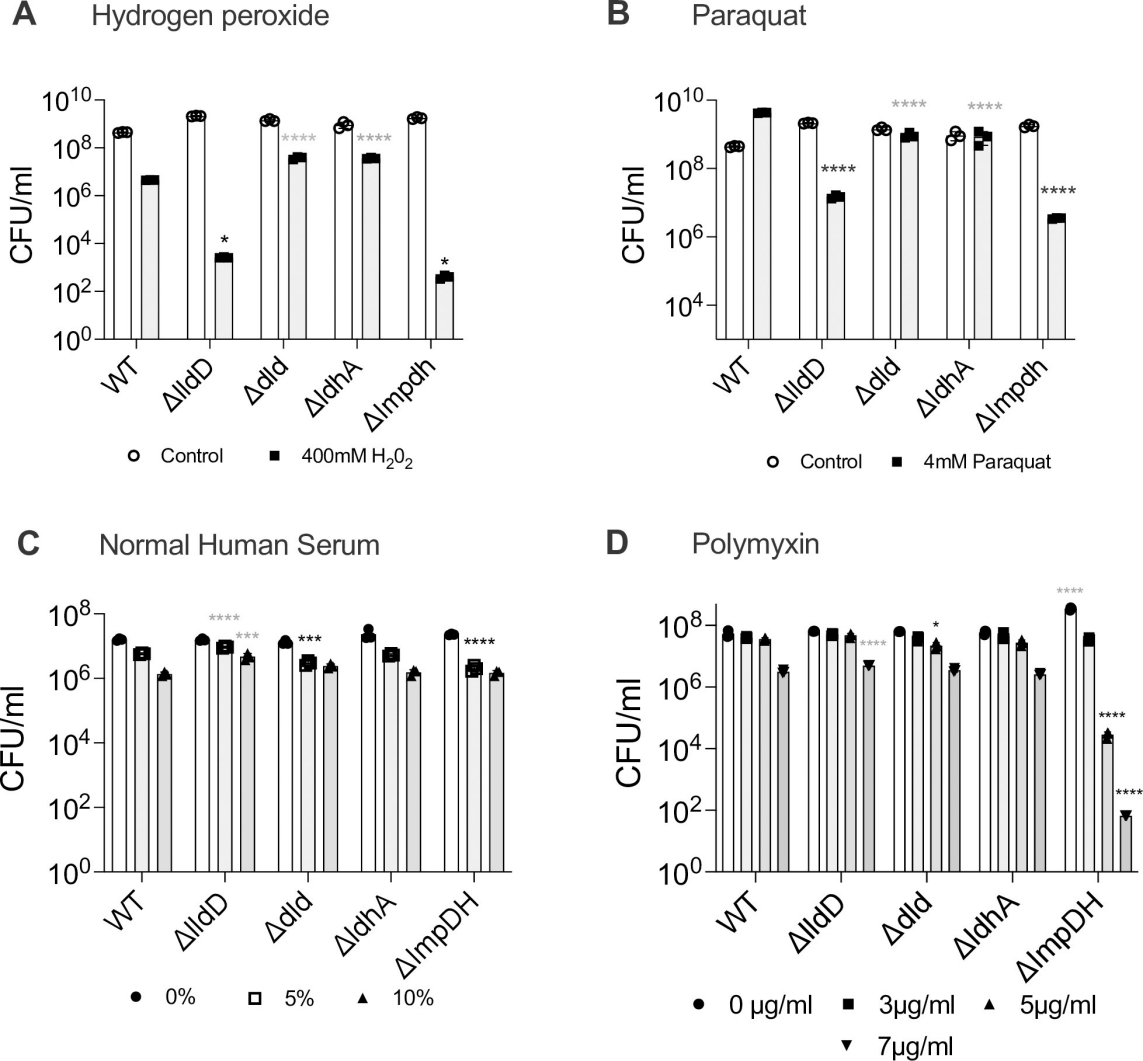
Susceptibility of Hi2019^WT^ and Hi2019 LDH and ImpDH mutant strains to cell envelope and oxidative stress that may be encountered during infection. **A:** 400 mM hydrogen peroxide, **B:** 4 mM paraquat, **C:** Normal human serum (5% and 10%) **D:** Polymyxin B (3 μg/ml, 5 μg/ml, and 7 μg/ml). Bacteria suspensions were treated with the stressor for 1h before being serially diluted except when human serum was used (Panel C) where a 45 minute exposure time was used. All data shown are averages ± STDEV of three replicates; * p<0.05, *** p<0.001, **** p<0.0001; Statistical testing used One-way ANOVA and either Benjamini -Krieger post-hoc test (Panel A) or Dunnet’s post-hoc test (Panels B, C, D). Statistical significance is reported relative to the WT data for the same condition where a grey asterik/s indicates the mutant strain is more resistant than the WT and black asterik/s indicate the mutant strain is less resistant than the WT.

Metabolic defects not only change the flux of metabolites through specific pathways, but also alter the functioning of the bacterial cell, which might contribute to the attenuation observed for the Hi2019^Δ*lldD*^ and Hi2019^Δ*ImpDH*^ strains during infection. We therefore tested whether the LDH and ImpDH mutations were associated with changes in strain resistance to oxidative stress and cell envelope integrity. Unexpectedly, both the Hi2019^Δ*lldD*^ and Hi2019^Δ*ImpDH*^ strains were significantly more susceptible than the wild-type to exposure to hydrogen peroxide and paraquat (Fig 7A and 7B). This specific defect may have relevance during infection where both hydrogen peroxide and superoxide are produced by host epithelial and immune cells, and this susceptibility may enhance strain clearance. In contrast, changes in the resistance to envelope stress caused by exposure to human serum or polymyxin were less drastic for most strains. Following exposure to human serum, the Hi2019^Δ*dld*^ and Hi2019^Δ*ImpDH*^ mutants showed a slight increase in sensitivity, while Hi2019^Δ*lldD*^ was somewhat more resistant than the wild-type strain (Fig 7C). Similar data were obtained for resistance to polymyxin B, except that here Hi2019^Δ*ImpDH*^ showed increased susceptibility to polymyxin killing (Fig 7D).

Despite the sometimes drastic changes in oxidative stress resistance and polymyxin resistance in the different mutant strains, an analysis of the metabolic endproducts produced by the LDH mutant strains following growth on glucose-containing CDM medium only revealed minor changes (S6 Table). All strains produced acetate, succinate and formate in similar proportions, however, while results for Hi2019^WT^ and Hi2019^Δ*ldhA*^ were essentially identical, both Hi2019^Δ*dld*^ and Hi2019^Δ*lldD*^ appeared to use increased amounts of glucose, which in Hi2019^Δ*lldD*^ was paired with a slight reduction in acetate production (S6 Table). This suggests that while the primary role of LldD is in L-lactate utilization, removal of either of the two membrane-bound lactate dehydrogenases may affect energy conservation and redox balancing, leading to a higher use of glucose, which is also in keeping with results obtained during the initial characterization of the strains.

### The NTHi metabolic end product acetate alters host cell immune responses

Acetate is a major metabolic endproduct of NTHi metabolism under all condition we have tested [32,34], and during tissue cell infection can accumulate to high concentrations (5–8 mM) (Fig 2A). SCFAs such as acetate have been linked to both pro- and anti-inflammatory effects on human tissue cells, depending on the context in which they are produced [51,52], and we hypothesized that the presence of acetate in infection assays could alter the host immune response. To simulate an established infection, we added 7 mM acetate to 16HBE14 infection assays and used live as well as heat-killed NTHi that provide only antigenic stimulation to identify effects that require live bacteria.

**Fig 8:**
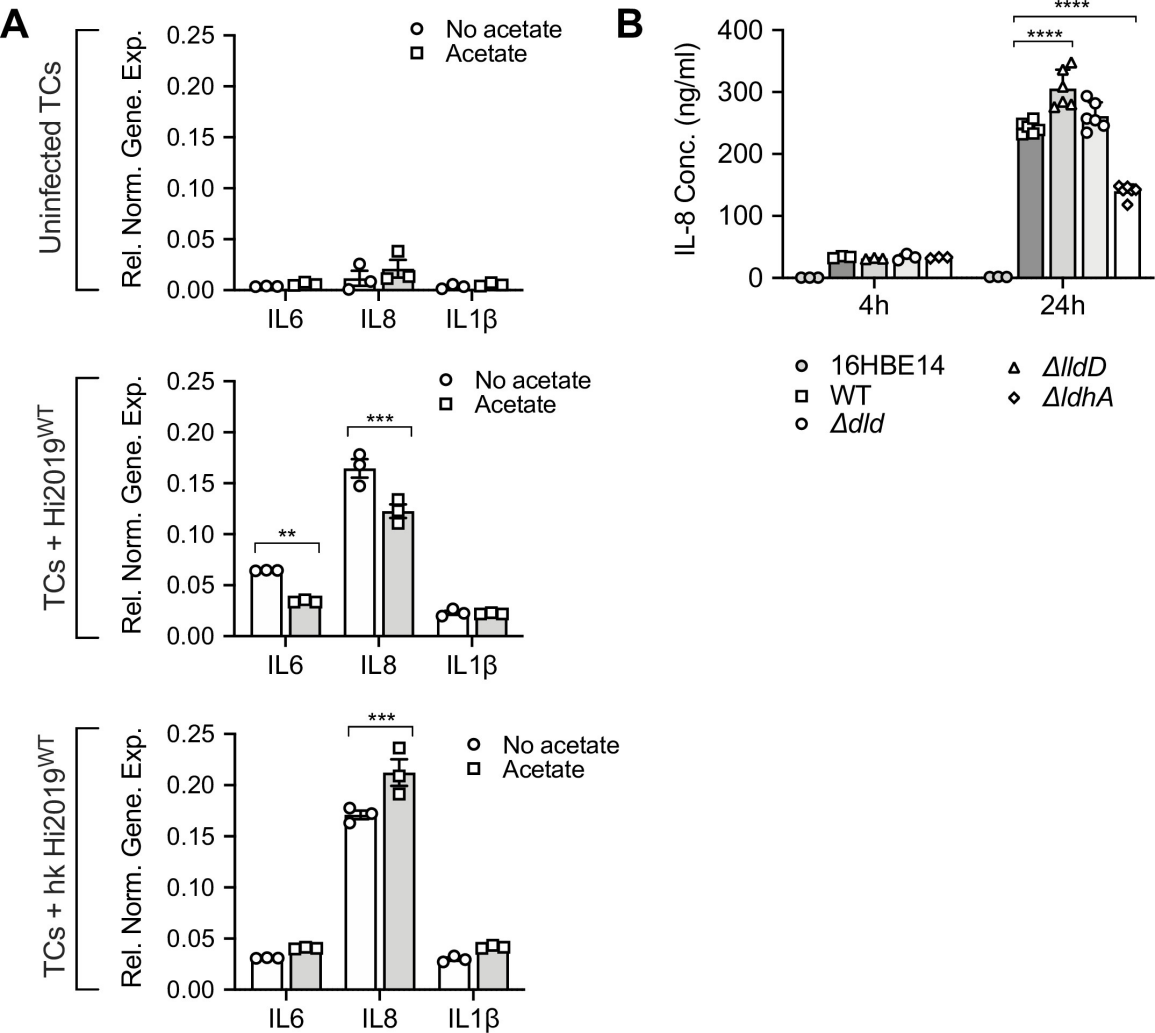
Interleukin (IL-6, IL-8, and IL-1β) production in Hi-infected 16HBE14 cells. **A:** Effects of externally added acetate (7 mM) on the expression of interleukin genes (IL-6, IL-8, and IL-1β) in 16HBE14 infected with live or heat-killed Hi2019. Hi2019 prepared after 18h growth in sBHI. Gene expression was normalised using the ACTB gene, three replicates were used for each data point. Statistical significance is reported relative to the control for each interleukin. **B:** IL-8 levels in culture supernatants of 16HBE14 infected with Hi2019^WT^ and lactate dehydrogenase mutant strains for 4 h (n = 3) and 24 h (n = 6) post-infection. IL-8 levels were detected using a human IL-8 ELISA assay (Sigma). Statistical significance is reported relative to the WT infection. All data are shown as averages ± STDEV; * p<0.05, ** p<0.01, *** p<0.001, **** p<0.0001; Statistical testing for each group used 2-way ANOVA (factors: genes, acetate treatment, Panels A, B, C; Panel D: strain & time) with Dunnett’s (Panels A, B, C) or Tukey (Panel D) post-hoc test.

As expected, the presence or absence of externally added acetate did not result in statistically significant (p<0.05) changes in IL-6, IL-8 and IL-1β gene expression in uninfected tissue cells (Fig 8A), and we also observed the typical increase in transcription of IL-6, IL-8 and IL-1β genes following infection with live Hi2019^WT^ (17-, 14- and 6.5-fold compared to the uninfected tissue cells) (Fig 8A). However, when acetate was included in the Hi2019^WT^ infection assay, an anti-inflammatory effect was observed that resulted in a 47% (2-Way ANOVA, p = 0.0021) and 26% (2-Way ANOVA, p = 0.0001) reduction of IL-6 and IL-8 expression, while IL-1β expression was essentially unchanged.

Exposure of 16HBE14 to heat-killed NTHi, which provide only antigenic stimulation, also increased expression levels of IL-6, IL-8 and IL-1β between 8.3- and 14.7-fold compared to the uninfected 16HBE14 when no acetate was added. In this system, however, added acetate exerted a pro-inflammatory effect, with increases in the expression levels of all three monitored interleukins, even though only the 24% increase in IL-8 expression was statistically significant (2-Way ANOVA, p = 0.0007) (Fig 8A).

While live NTHi can access both extra- and intracellular niches during infection of 16HBE14 cells, heat-killed NTHi are not able to establish actively growing extra- or intracellular communities and are unable to interact with the host on a metabolic level. Our data then suggest that live NTHi actively modulate the immune response of the host cell in the presence of acetate, which may require specific metabolic interactions or the ability to access the intracellular niche, where production of acetate by live NTHi might directly affect host cell metabolism and inflammatory responses. To test whether intracellular colonization plays a role in the observed anti-inflammatory effects, we determined IL-8 concentrations in 16HBE14 culture medium following infection with Hi2019^WT^ and the Hi2019^Δ*lldD*^ strain. Hi2019^Δ*lldD*^ showed a defect in access to and/or survival in the intracellular environment in this infection model and should, if intracellular localization of NTHi contributes to the anti-inflammatory effect, show an increase in IL-8 production. In keeping with this, IL-8 levels in Hi2019^Δ*lldD*^ samples were elevated by 25% compared to Hi2019^WT^ (2-Way ANOVA, p<0.0001), while for all other lactate dehydrogenase mutant strain levels were similar or slightly reduced (10–15%) compared to the Hi2019^WT^ (Fig 8B).

## Discussion

Despite extensive studies of *H*. *influenzae* genomics and modelling of its metabolic network [53,54], comparatively little is known about carbon and energy sources required by *H*. *influenzae* strains during growth in the host. This is in contrast to several other facultatively and obligately intracellular human pathogens such as *E*.*coli*, *Listeria*, *Brucella* and *Legionella*, where glucose, glycerol and amino acids have been identified as supporting intracellular growth while *Mycobacterium tuberculosis* (Mtb) has been shown to rely mostly on cholesterol degradation for intracellular survival [31,55–58].

Here we have undertaken a comprehensive survey of the NTHi carbon, nitrogen and phosphorous source profiles that revealed that they are extremely limited and highly adapted to the human respiratory tract. Lactate, nucleosides, pentose and triose sugars were highly used by several unrelated Hi strains. In contrast, growth on most of the substrates traditionally used in Hi growth media and metabolic models such as glucose, fructose, glutamate and sialic acid was only average to low (Fig 1A and S1 Table). The relevance of our analysis is highlighted by comparison to a recent study of the metabolome of human nasal and bronchial epithelia. Of the Hi C, N, and P sources we identified here, the majority (C: n = 11; N: n = 8; P: n = 9) occur naturally on human nasal and bronchial epithelial surfaces [59], emphasizing the specific adaptation of Hi metabolism to the human respiratory tract. Additionally, several metabolites showed differential distribution between nasal and bronchial epithelia, with inosine being more prevalent on bronchial epithelia while more lactate was recovered from nasal samples [59].

**Fig 9:**
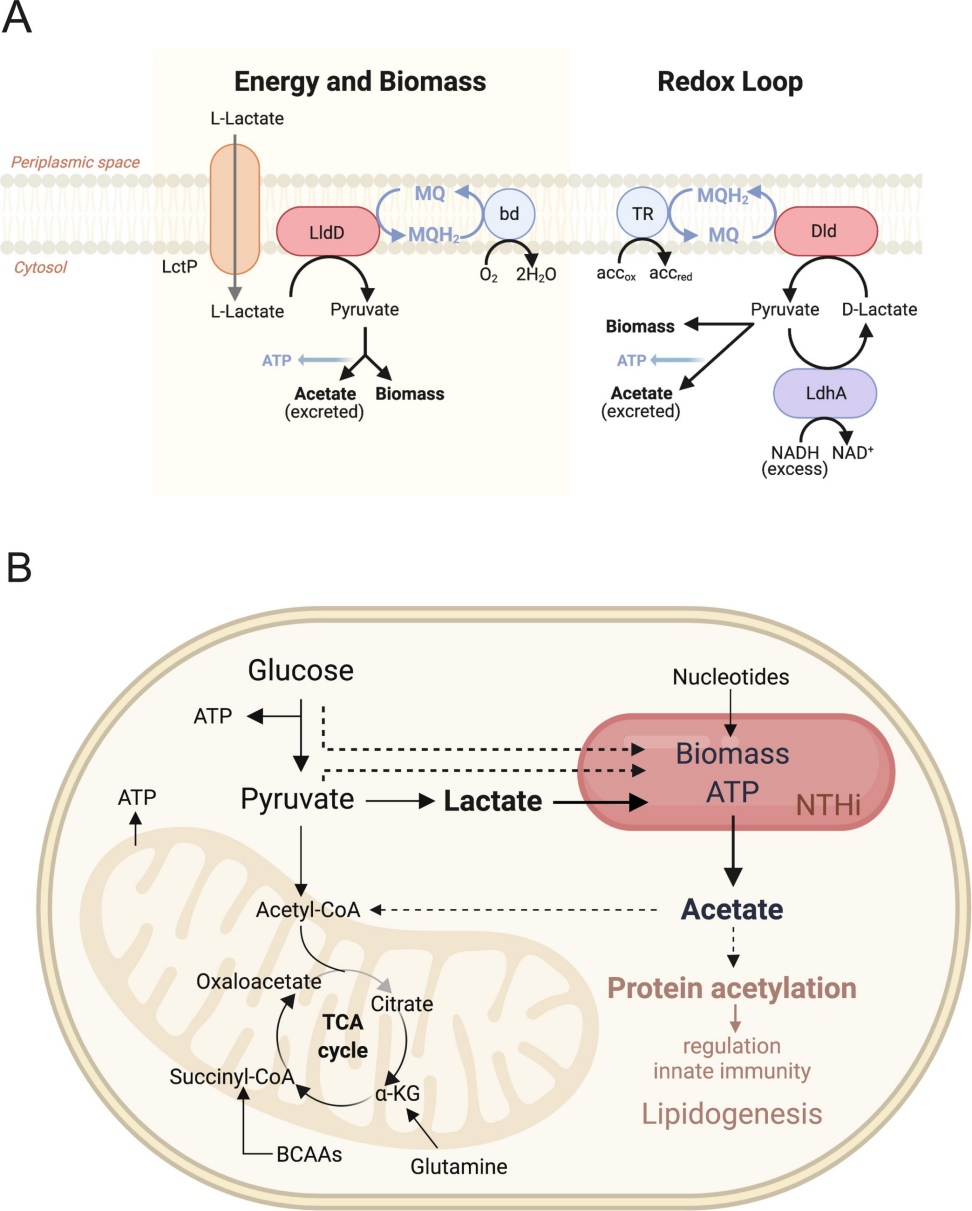
Models of NTHi lactate dehydrogenase functions and NTHi carbon source utilization during tissue cell infection. **A:** Functions of lactate dehydrogenases in NTHi strains. bd = bd-oxidase, TR = terminal reductase, MQ/MQH_2_ –menaquinone/menaquinol **B:** Model of carbon sources used by NTHi strains during infection of human epithelial cells. Dashed arrows: proposed carbon flow; brown font: proposed cellular effects.

Access to highly used substrates is important for *H*. *influenzae* survival in contact with host cells as we have demonstrated here for L-lactate and guanine (Figs 4 and 5). In-depth analysis of Hi lactate utilization showed that the three lactate dehydrogenases found in ~90% of Hi strains serve two independent functions, namely biomass and energy generation via the LldD L-lactate dehydrogenase, and redox balancing via an intracellular D-lactate/pyruvate interconversion loop that involves the action of Dld and LdhA (Fig 9A). To generate biomass and energy, LldD converts L-lactate to pyruvate, which can be easily assimilated into biomass, or used to produce acetate, where the reaction of acetate kinase generates ATP (Fig 9A). The LldD reaction produces menaquinol, which can be re-oxidized by the bd-type cytochrome oxidase using oxygen as the electron acceptor or one of the alternative respiratory complexes present in Hi that operate with alternative electron acceptors under anaerobic/microaerobic conditions [34].

In contrast, the D-lactate dehydrogenases Dld and LdhA appear to be mostly involved in redox balancing under oxygen-limiting conditions, regardless of the carbon source present (Fig 9A). Under conditions of redox stress caused e.g. by over reduction of the NAD^+^/NADH pool, the Dld/LdhA redox loop enables a re-oxidation of NADH to NAD^+^ via the reaction of LdhA, leading to production of D-lactate from pyruvate. Instead of being excreted, D-lactate is converted back to pyruvate by Dld with the electrons being transferred to the menaquinone pool (Fig 9A). This combination of Dld and LdhA reactions thus allows redox balancing to occur via the respiratory chain while preserving a valuable carbon molecule, pyruvate, for use in energy or biomass generation (Fig 9A). This Dld-LdhA redox loop may be the reason why D-lactate is generally not excreted as a metabolic endproduct in NTHi, although we have recently shown that following extreme metabolic perturbances D-lactate production could be observed in NTHi [26,34]. An additional role for the Dld LDH could be the conversion of D-lactate formed by other reactions such as the breakdown of dihydroxyacetone-phosphate to methylglyoxal [60].

Despite appearing to be able to function independently, we observed some interdependence between the LldD and Dld/LdhA systems in enzymatic activities and respiratory capacity, potentially linked to changes in the cellular redox state. However, in infection assays, strong phenotypes were always associated with the ability of Hi to utilise L-lactate via LldD, which matches a previous study that associated defects in lactate uptake with reduced Hi virulence [61]. Unexpectedly, the mutation in the *lldD* lactate dehydrogenase was associated with defects in intracellular survival of NTHi in all three infection models we used. In the NHNE organoid model, LldD was required for long-term intracellular survival of NTHi in single infections, while in competitive infections Hi2019^Δ*lldD*^ was outcompeted by the wildtype strain both extra- and intracellularly, and during both initial infection phases and long-term persistence. While it seems likely that the inability to use L-lactate was the reason for the reduced fitness of the Hi2019^Δ*lldD*^ strain, the increased sensitivity of the strain to oxidative stress may have contributed to this phenotype, especially in the mouse infection model where immune cells are present. It is not clear why the *lldD* mutation would cause a sensitivity to oxidative stress as we did not detect decreases in cell envelope integrity (Fig 7), but the phenotype could be associated with changes in the cellular redox balance and a loss of energy generation via acetate formation from L-lactate.

A similar combination of respiratory L- and D-lactate dehydrogenases and a soluble LdhA D-lactate dehydrogenase appears to be conserved in several lactate-utilizing mucosal pathogens, including *Neisseria gonorrhoeae* [46]. This highlights the importance of these lactate utilization systems for bacterial virulence, however, there were several notable functional differences between lactate utilization in Hi and *N*. *gonorrhoeae*. The latter produces D-lactate as a regular metabolic end-product (~6 mM), and mutation of *ldhD* gene reduced gonococcal survival in both human neutrophils and cervical epithelial cells [46], while the equivalent *dld* gene did not appear to have a role in NTHi virulence in tissue cells or NHNE. An LldD-like enzyme was also required for maximal replication of *Mtb* in macrophages [62,63], but in this system higher concentrations of lactate could be inhibitory for *Mtb* growth, which was not the case for Hi (Fig 1).

A second enzyme with a key role in Hi virulence is IMPDH, which controls the cellular supply of guanine. IMPDH has been linked to decreases in virulence in various fungal as well as bacterial pathogens and has been postulated to be required for fast proliferation of pathogens [64–66]. In Hi the loss of IMPDH affected growth on minimal but not rich media *in vitro*, which is in keeping with the low availability of guanine in CDM medium. To the best of our knowledge, this is the first time that guanine biosynthesis has been shown to play a role in NTHi virulence. The reduction of Hi2019^Δ*ImpDH*^ extra- and intracellular cell numbers during single and competitive infection of NHNE indicates that in longer-term infection, guanine supplies became increasingly limiting, thus reducing survival and persistence of Hi2019^Δ*ImpDH*^.

Survival of intracellular pathogens in the host is closely linked to an ability to strike a balance between access to and consumption of metabolites for pathogen growth and the resulting perturbation of the host metabolic network that could lead to host cell death [55,56,58]. Utilization of L-lactate as a key carbon and energy source by Hi would likely only lead to small perturbations in carbon metabolic pathways in the host cell, as L-lactate is an endproduct of human cell metabolism and is only recycled in cases of low nutrient availability [67]. This strategy has been shown to exist in obligately intracellular bacteria and has been called ‘bipartite metabolism’ [68]. In contrast, glucose-utilizing intracellular pathogens such as *Brucella* and certain types of *E*.*coli* [55] directly compete with the host cell for the main carbon and energy source present in the human body.

Based on the data presented we propose a working model for metabolic interactions between intracellular Hi and host cells (Fig 9B) where Hi uses lactate as a main carbon source and derives ATP mostly from substrate-level phosphorylation during the production of acetate, which is released into the host cell cytoplasm, likely in significant amounts. During intracellular growth, Hi might also access pyruvate and possibly some glucose present in the host cell [32,34] (Fig 9B).

The production of acetate as a metabolic endproduct by Hi during co-culture with human cells is significant as it appears to contribute to a reduction in the host immune response to Hi. Acetate is a natural metabolite in human cells that can arise either during growth stages with high glycolytic rates or as a result of stress and nutrient starvation and can re-enter the host nutrient pool in the form of acetyl-CoA [69,70]. High acetyl-CoA concentrations can drive lipogenesis as well as general protein and histone acetylation, which affects gene expression and cell signalling [67,71] (Fig 9B). Our data indicate that acetate production by NTHi contributes to a dampening of the host immune response. As this effect was only observed for live NTHi, we suggest that it may be mediated either by metabolic interactions between NTHi and the host cell and/or by intracellular populations of NTHi. Some evidence for an effect of the latter comes from the higher production of IL-8 after infection with the Hi2019 Δ*lldD* mutant strain that is impaired in its ability to invade 16HBE14 cells (Fig 8B). Being able to reduce the host inflammatory response would promote the long-term persistence that is characteristic for many Hi mediated infections, but has not previously been linked to metabolic endproducts such as acetate as effectors.

Our work thus provides first comprehensive insights into Hi nutritional requirements during infection and metabolic processes relevant for intracellular growth, including the ability of metabolic endproducts to reduce the host cell immune responses to infection. NTHi is known to contribute to and exacerbate chronic respiratory tract infections, and its ability to survive in contact with host cells using lactate as a main carbon source while at the same time modulating the host cell immune response via the production of acetate adds important new mechanistic details to our understanding of Hi survival as a commensal and during exacerbations of chronic respiratory diseases.

## Methods

### Ethics statement

Experimental animal procedures were carried out in strict accordance with the recommendations in the QLD Animal Care and Protection Act (2001), and the Australian Code of Practice for the Care and Use of Animals for Scientific Purposes, 8^th^ edition. Protocols were approved by the Animal Care and Ethics Committees of QIMR Berghofer and the University of Queensland (QIMR/050/19). Human nasal cells were sampled and donated by healthy donors at the Child Health Research Centre, UQ, Brisbane (UQ Ethics approval #2017000520).

### Bacterial strains and growth conditions

*E*. *coli* strains (S7 Table) were cultured using LB medium [72]. NTHi 2019 [38] and strain derivatives (S7 Table) were cultivated using chemically defined growth medium (CDM, carbon sources: 10 mM D-glucose, 25 mM D-, L- or DL-lactate) [73] or supplemented brain heart infusion (sBHI) broth or agar as in [74]. ‘Complete’ CDM contained 10 mM glucose, 7.5 mM inosine and 1 mM pyruvate as carbon sources. Antibiotics (*Ec/*Hi: kanamycin 100/50 μg/ml, spectinomycin 50/20 μg/ml, *Ec*: ampicillin 100 μg/ml) or guanine (0.125–1 mM guanine, standard: 1 mM) were added where required.

NTHi growth experiments used microtitre plates (200 μl CDM medium/well) with three biological replicates/strain and 3 technical replicates/biological replicate at 37°C, 200 rpm using a Clariostar multimode plate reader (BMG LabTech). Gas-phases used: aerobic (20% O_2_, 0% CO_2_), microaerobic (2.8% O_2_, 5% CO_2_), anaerobic (0% O_2_, 5% CO_2_).

### Bactericidal assays

NTHi cell material freshly grown on sBHI plates was resuspended to an OD_600nm_ of 1.0 in 1 x sterile Phosphate Buffered Saline (PBS). 900μL of the bacterial culture were then combined with 100 μL of a freshly prepared 10 x stock of the test compound, incubated at room temperature with gentle orbital shaking for 1 h followed by immediate serial dilution (up to 10^−7^) in BHI and plating on sBHI plates. Assays used 400 mM H_2_O_2_, 4 mM paraquat or 1–7 μg/mL polymyxin B. Normal human serum-mediated killing assays were performed as previously published [75]. In brief, Hi2019 strains were grown to mid log-phase (OD_600nm_ of 0.5–0.6) and then harvested (800 x *g*, 10 min, 20°C), and resuspended in 1x PBS supplemented with 0.5% BSA, 5 mM MgCl_2_ & 1 mM CaCl_2_. 2 x10^7^ bacteria/mL were incubated with 0–10% serum for 45 min at 37°C followed by serial dilution. Each susceptibility assay was repeated on three independent days, on each day three biological replicates were used for each strain.

### Omnilog Phenotypic Microarray

NTHi cell material from a freshly sBHI plate, following overnight microaerophilic growth, was resuspended into 2-3ml of IF-0a or IF-10b followed by the PM (12x) additives as required (S8 Table). The turbidity of the inoculum was adjusted to 65%T with uninoculated media as the blank (100% T). 100μl of the 65%T inoculated 1xPM solution were added to each well of the corresponding Biolog PM plate. Plates were loaded into the Biolog incubator (37°C) and readings taken every hour for 48 h.

### Molecular and biochemical methods

Standard methods were used throughout [72]. Hi2019 genomic DNA was isolated by the Purelink Genomic DNA Mini Kit (Invitrogen), and oligonucleotides (S9 Table) were supplied by Integrated DNA technology. Plasmid isolation and PCR product clean up used the PureLink Quick Plasmid Miniprep Kit (Invitrogen) and Wizard SV Gel and PCR Clean-up System (Promega). Restriction enzymes and Ligase were from NEB and Life Technologies. Protein concentrations were determined using the BCA-1 kit (Sigma Aldrich).

### Construction and complementation of Hi2019 mutant strains

Plasmids (S7 Table) for creating single gene knock out mutations were constructed using a two-stage process where either a single gene fragment (*lldD*, C645_RS09865, WP_005657875.1) or two gene fragment (*dld*, C645_RS09380, WP_046067851.1; *ldhA*, C645_RS00700, WP_005672300; *guaB*, C645_RS01605, WP_005660592.1) were cloned into pGEMT-Easy (Promega) or pBluescriptIISK (Stratagene), respectively, yielding pGEM-Hi*lldD*, pBlu-Hi*ldhA*, pBlu-Hi*dld*, and pBlu-Hi*ImpDH*. Suitable restriction enzyme sites were used to insert a kanamycin (*kan*) cassette, amplified from pUC4K [76], to yield pGEM-Hi*lldDkan*, pBlu-Hi*ldhAkan*, pBlu-Hi*dldkan*, and pBlu-Hi*ImpDHkan*. Hi2019 was transformed with these plasmids, to generate Hi2019^Δ*lldD*^, Hi2019^Δ*dld*^, Hi2019^Δ*ldhA*^, and Hi2019^Δ*ImpDH*^ using the method of [77]. Mutant strains were complemented with p601.1sp containing a functional copy of the gene and associated promoter regions, cloned using an *XmaI* site (S7 Table). Complementation plasmids were transformed, as described above, into the appropriate mutant strain. All mutant and complemented strains were verified by PCR and enzyme assays.

### Preparation of cell extracts and subcellular fractions

Cell-free extracts of NTHi were generated using either BugBuster Mastermix (Novagen) according to the manufacturer’s instructions (750 μl were used for pellets from 20 mL culture) or 3 rounds of bead beating (HT Mini OPS Diagnostics with 100 mg beads) for 30 seconds with extracts cooled on ice between rounds. Soluble protein and membrane fractions were separated by ultracentrifugation (Optima XPN–100, 50.2 Ti rotor, Beckman Coulter) at 110,000 x *g* for 2 h at 4°C. Membranes were resuspended in 0.5 ml 10 mM Tris-Cl (pH7.5).

### Enzyme assays

All assays were carried out at 37°C using a Cary60 spectrophotometer (Agilent Technologies) and monitored at 570 nm (MTT and DCPIP-based assays) or 340 nm (NADH-based assays). MTT-based reactions contained 10 mM 3-(4,5-Dimethyl-2-thiazolyl)-2,5-diphenyl-2H-tetrazolium bromide (MTT) (ℇ_570nm_ = 17 mM^-1^ cm^-1^) and 10 mM Tris-Cl (pH 7.5). DCPIP-based reactions contained 0.1 mM 2,6-dichloroindophenol sodium salt hydrate (DCPIP) (ℇ_570nm_ = 21 mM^-1^ cm^-1^ [78]), 20 mM potassium phosphate buffer (pH 7.4), 2 mM MgCl_2_, and 0.25 mM phenazine methosulfate (PMS). Both MTT and DCPIP-based assays contained 0.0028–16 mM (std. conc.: 2.5mM) L- or D-lactate, and 0.2–0.5 mg/ml CFE. NADH-based reactions contained 0.0035–0.6 mM (standard conc.: 0.06 mM) NADH (ℇ_340nm_ = 6.2 mM^-1^ cm^-1^ [46]), 50 mM Tris-Cl (pH7.5), 0.2–0.5 mg/ml CFE, and either 0-50mM (std. conc.: 10 mM) sodium pyruvate or 5 mM of either D- or L-lactate. Specific activities for all assays are given as μmol of substrate reduced/oxidised per min (U) and mg of protein present in the assay.

### Whole-cell respiration assays

These assays were performed essentially as in [46] using a Hansatech oxygen electrode and washed. Biological triplicates of NTHi strains were cultured microaerobically in CDM, harvested (2800 x *g*, 10 min, 4°C) at mid-log phase, and washed 2x with 1xPBS (pH 7.4). Assays used 0.5–1 mg/ml protein with either 10 mM D-glucose, L-lactate, or D-lactate to start respiration.

### NTHi host cell adherence and invasion assays using cultured tissue cells and NHNE

Human bronchial epithelial 16HBE14 cells [79] were cultured using Minimal Essential Medium (MEM) with 10% FBS and NTHi adherence and invasion assays performed as in [74]. Normal Human nasal epithelia (NHNE) were prepared as in [80] from primary nasal cells sampled and donated by healthy donors at the Child Health Research Centre, UQ, Brisbane (UQ Ethics approval #2017000520). Seven days prior to infection, the NHNE were transferred to steroid- and antibiotic-free PneumaCult-ALI media (Stemcell). NTHi for infection were resuspended in 1×PBS and used at MOI 10:1. Uninfected control replicates were exposed to an equal amount of sterile PBS. After infection for 24 h at 37°C, 5% CO_2_ the inoculum was removed by washing with 1×PBS, and the basal medium replaced. NHNE apical surfaces were washed with 100 μl PBS every 2 days, while the basal medium was replaced every 2 days. Lysates were generated by 10 min incubation of NHNE with 1% saponin at 37°C. Intracellular bacteria CFU were determined after treating the NHNE surface with gentamycin (100 μg/ml) for 1 h at 37°C. Gentamycin was then aspirated and the apical and basal compartments washed 5× in 200 μl and 1ml PBS, respectively before lysis. Bacterial loads in lysates were determined by plating serial dilutions on sBHI agar. NHNE transepithelial electrical resistance (TEER) was measured every 2 days using a Millicell ERS-2 Volt-ohm meter. For competition assays, NHNE were infected with an equal mixture of Hi2019^WT^ and one mutant strain (MOI 10:1) and bacterial loads analyzed as described above, except that serial dilutions were plated on sBHI to determine total bacterial numbers as well as sBHI with 20 μg/ml kanamycin to determine the load of NTHi carrying a mutation. Hi2019^WT^ cell numbers were determined by subtracting CFU/ml of kanamycin-resistant (mutant) NTHi from total NTHi CFU/ml for the same time point and used to compute fold-changes. Each infection and co-infection used two biological replicates for each strain or strain combination. For each biological replicate, three technical replicates were used to determine CFU/ml.

### *H*. *influenzae* mouse model of lung infection

For NTHi pulmonary infection, a mouse model described previously by [81] was used. NTHi strains for infection were grown on sBHI plates for 16 h at 37°C with 5% CO_2_. BALB/c female mice (5 to 6 weeks old) were inoculated intranasally with 30μl of a bacterial suspension containing 10^7^ CFUs. Groups of 5 mice were euthanized and necropsied at 0, 6 h, 24 h, 48 h and 72 h. To quantify bacterial recovery, lungs were aseptically removed. Lung tissue was either snap-frozen in liquid nitrogen for RNA isolation or homogenized in 1 mL 1× PBS, serially diluted in the same buffer followed by plating of dilutions on sBHI plates. Bronchioalveolar lavage fluid (BALF) was also collected and CFU/ml determined. CFUs per lung were calculated as in [82]. Giemsa staining of BALF was used to stain immune cells as per the manufacturer’s (Sigma-Aldrich) instructions. Statistical comparisons of mean CFU/lung and in BALF were performed using two-tailed t-tests as integrated into the Prism 9 software package.

### ELISA assays

Cell culture supernatants collected from uninfected and Hi infected 16HBE14 cultures were used to determine host IL-8 production (RAB0319 kit, Sigma-Aldrich), according to manufacturer’s instructions. Two biological replicates of each sample at 24 h, with triplicate technical replicates, were used in the assay. ELISA assays (mouse TNFα & IL1β) of mouse BALF used uncoated ELISA assays (Thermofisher) according to the manufacturer’s instructions. Data were collected for all mice in each treatment group. Analyses used 2-way ANOVA with Dunnett’s (Tissue cell supernatant) or Fisher LSD (mouse BALF) post-hoc test.

### Host cell challenge with NTHi metabolites assay

Human bronchial epithelial 16HBE14 cells [79] were cultured as in [74] in 24 well plates with 1x10^5^ cells/well. NTHi for infection were grown microaerobically in sBHI and 5 ml of this culture was heat-killed (1.5 h at 70°C). Both viable and heat-killed NTHi was diluted to OD_600nm_ 0.05 in sMEM. 1 ml viable, heat-killed, and uninfected sMEM, both with and without 7 mM acetate, was added to 16HBE14 cells in triplicate. Hi infected and uninfected cells were incubated for 24 h at 37°C with 5% CO_2_ before the supernatant was removed and 16HBE14 RNA was preserved using Trizol (Life Technologies).

### RNA isolation, cDNA preparation, and qRT-PCR

RNA from Hi, cultured AE, MA, and AN with 25 mM DL-lactic acid and 10 mM glucose, was preserved and isolated with 1 ml stop solution (200 mM Tris-Cl, 20 mM EDTA, 20 mM sodium azide, pH 8.0) (1:20) and 0.5 ml TRIzol (Life Technologies). 16HBE14 RNA was preserved and isolated with 1ml stop solution and 0.3 ml TRIzol per 24-well plate well. For RNA isolation from mouse lung tissue the snap-frozen lung tissue was homogenized in 1 ml Trizol (Life Technologies), followed by centrifugation to remove large cellular debris and RNA isolation as per the manufacturer’s instructions. RNA was isolated for 3 mice in each treatment group. All RNA samples were DNAse-treated and quantified as in [34]. Hi and 16HBE14 cDNA was synthesised from 250 ng and 500 ng, respectively, of RNA using the Superscript IV VILO Mastermix (Life Technologies) and RNAsin RNAse inhibitor (Promega). Mouse cDNA was prepared using the Lunascript RT SuperMix kit (New England Biolabs) and 500 ng RNA. qRT-PCR reactions were set up and analysed as in [34]. Reference genes used included *16S* and gyrase (*gyrA*) genes for Hi reactions and β-actin (*ACTB*) for 16HBE14 and mouse cDNA reactions.

### NMR metabolomics and HPLC-based metabolite detection

1 ml samples of culture medium supernatant were collected either after 4 h and 24 h from 16HBE14 tissue cell cultures with or without Hi2019 infection or from supernatants of bacterial cultures grown to stationary phase. All samples were preserved and prepared for ^1^H-NMR analysis as in [34] in 3 mm NMR tubes. Proton NMR spectroscopy was performed on a Bruker AV900 900MHz spectrometer and one–dimensional proton spectra were measured essentially as in [83] but using 256 scans. 1D spectra were processed using Topspin 3.0 (Bruker Biospin) and Chenomx NMR Suite 8.2 (Chenomx Inc., Edmonton, Canada) as in [34]. Detection of acetate, succinate and formate by high-performance liquid chromatography (HPLC) was carried out as in [26] using supernatants from cultures grown on defined medium with either glucose (10 mM) or lactate (4 mM) and inosine (7.5 mM) as the carbon sources. Metabolite detection used an Ultimate 3000 HPLC system (Thermofisher Scientific) equipped with a UV detector and an RI detector (Shodex RI-101). Samples were separated (total runtime: 20 min) on a Metab-AAC column (300 x 7.8 mm; Isera, Dueren) at 30°C, a flow rate of 0.8 ml/min and a running buffer of 5 mM sulfuric acid. Data analysis used Chromeleon (Thermofisher Scientific).

### Bioinformatic analyses

Genes related to lactate metabolism in Hi strains were identified using BLASTP [84] with the listed reference protein sequences (S4 Table) and cut-off criteria (S4 Table). Properties of Hi LldD, Dld, and LdhA were analysed using SignalP3.0, TatP1.0, TMpred, TMHMM, and Compute pI/MW, and Pfam [85–88].

### Statistical analyses

Data are presented as means ± SD. To determine differences between groups, depending on the type of data and comparison required, one- or two-way ANOVAs or two-tailed t-tests were performed. Statistical analyses were performed using the GraphPad Prism 9 software (GraphPad Software Inc., CA, USA). A p<0.05 was generally considered statistically significant, statistical tests were chosen depending on the structure of the data under analysis.

## Supporting information

S1 Fig Growth of NTHi strains in the presence of osmotic stressors, phenotypic microarrays (PM09-PM10 plates) **A**: sodium chloride, **B:** potassium chloride, **C:** sodium phosphate, **D:** ammonium sulfate, **E:** sodium nitrite, **F:** sodium formate, **G:** ethylene glycol.
(PDF)

S2 Fig Characterization of Hi2019^WT^ lactate utilization. **A:** Metabolites produced by strains Hi2019 (solid colour) and R2866 (diagonal stripes) during growth on CDM containing glucose & lactate (Glc/L-Lac– 10 mM glucose, 4 mM L-lactate) or lactate & inosine (L-Lac/inosine– 4 mM L-lactate, 7.5 mM inosine). Metabolites were analysed by HPLC. Formate was only detected in samples of Hi2019 following growth with glucose as te carbon source at a concentration of 0.8 ± 0.1 mM and is not shown in the graph as a result. **B:** Lactate dehydrogenase activities in Hi2019^WT^ following growth on CDM with lactate or glucose as the carbon source. Assays were conducted following growth under aerobic, microaerobic and anaerobic conditions **C:** gene expression of genes involved in lactate and inosine metabolism in Hi2019 following growth on CDM containing either glucose and L-lactate as the carbon source. **D:** Distribution of Dld and LldD activity in Hi2019 cell membranes (Mem.) and soluble (Sol.) cell extracts.
(PDF)

S3 Fig Verification and complementation of Hi2019 lactate dehydrogenase mutant strains using enzyme assays. Each assay was conducted using the Hi2019^WT^ strain, the LDH-mutant strain and the complemented strain following growth on CDM with glucose, as some strains (D*lldD*) were unable to grow on lactate containing media.
(PDF)

S4 Fig Macrophage and Neutrophil cell counts in BALF from mice infected with Hi2019^WT^ or Hi2019^Δ*lldD*^. Immune cells were detected using a Giemsa stain **A:** macrophage cell counts, **B:** neutrophil cell counts. Statistical analyses used 2-way ANOVA (Sidak post-hoc test), **** p<0.001; *** p<0.001. a p<0.05 was considered statistically significant.
(PDF)

S5 Fig Lung associated immune responses in mice infected with *H*. *influenzae* wildtype (white) and *lldD* mutant (grey) strains. **Panel A:** Expression of genes encoding key cytokines (IL-6, TNFa, IL-1b) and innate immunity signalling molecular (BIRC3) in mouse lung tissue during infection. Gene expression levels shown are for 3 mice per timepoint and are expressed relative to ACTB. **Panel B:** Cytokine levels in BALF from infected mice. Levels of TNFa and IL-1b shown are averages of 5 mice per timepoint. Statistical testing used 2-WAY ANOVA (Fisher’s LSD post hoc test), *—p<0.05, **—p<0.01, ***—p<0.0001.
(PDF)

S1 Table Omnilog Phenotypic microarray data PM01-PM10 for five NTHi strains (Hi2019, 86-068NP, C188, R2866, Hi535). Data shown are final absorbances after 24h of growth. PM plate numbers, well numbers as well as the compound contained in each well are given in the individual spreadsheets. PM9-10 are shown separately as they test resistance to osmotic and other environmental stressors.
(XLSX)

S2 Table Metabolites detected by ^1^H-NMR in spent culture medium of Hi2019^WT^ infected and uninfected 16HBE14 tissue cells. Metabolites in brackets have extensive overlap with other NMR signals. MEM–Minimal Essential Medium.
(PDF)

S3 Table Growth rates of Hi2019 WT and lactate dehydrogenase mutant strains on CDM containing 10 mM glucose, 25 mM D/L lactate or 25 mM L-lactate. None of the strains was able to grow with D-lactate as the substrate. Shading: highlights conditions with altered growth rates compared to the WT strain.
(PDF)

S4 Table Proteins involved in lactate conversions in *Haemophilus influenzae* (Hi lactate metabolism tab). Protein sequences from *E*. *coli* K12, *H*. *influenzae* 2019 and *N*. *gonorrheae* FA1090 used in BLASTP searches and cut-off parameters used for analysis of lactate utilization systems in *H*. *influenzae* (taxid 727) are given under ’Ref Sequences’.
(XLSX)

S5 Table Kinetic parameters of *H*. *influenzae* 2019 lactate dehydrogenases. LldD and Dld were assayed with both MTT and DCPIP as artificial electron acceptors, LdhA with pyruvate/NADH as substrates. No LdhA activity was detected with L- or D-lactate/NAD as substrates. *k*_M_ and V_max_ were determined by direct non-linear fitting of data to the Michaelis-Menten equation, values were determined using three biological replicates, error shown: standard deviation.
(PDF)

S6 Table Key metabolic endproducts produced by Hi2019 lactate dehydrogenase mutant strains. All substrate concentrations are given in mM. Bold font–reduced endproducts concentrations in the *lldD* mutant strain.
(PDF)

S7 Table Bacterial strains and plasmids used in this study. (PDF)

S8 Table Media additives used in Omnilog experiments (source: BIOLOG, fastidious organism protocol). Table adapted from Biolog Inc, Hayward, CA, USA (https://www.biolog.com/products-portfolio-overview/phenotype-microarrays-for-microbial-cells/).
(PDF)

S9 Table Oligonucleotide primers used in this study. (PDF)
