# Supplementary figures and images for: Access to highly specialized growth substrates and production of epithelial immunomodulatory metabolites determine survival of Haemophilus influenzae in human airway epithelial cells
Source: PLoS Pathog. 2022 Jan 27;18(1):e1010209. doi: 10.1371/journal.ppat.1010209 (PMC8794153; doi:10.1371/journal.ppat.1010209)

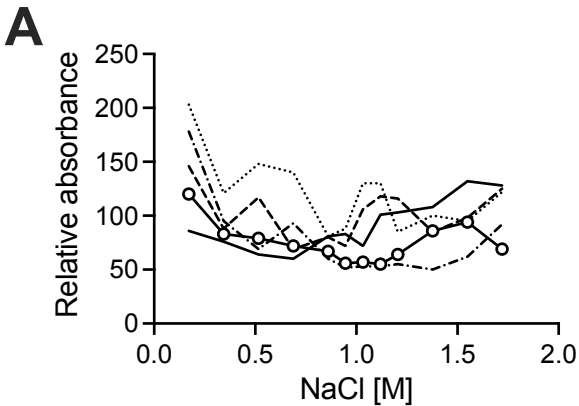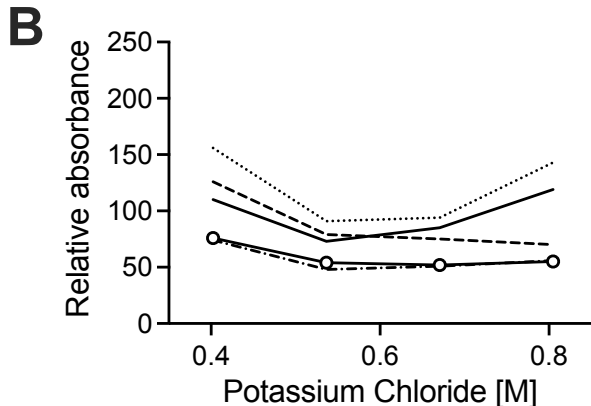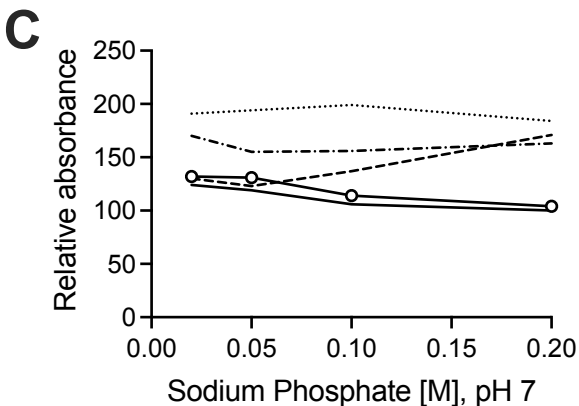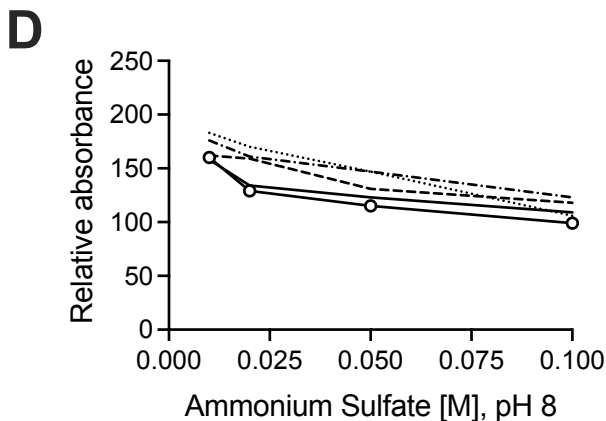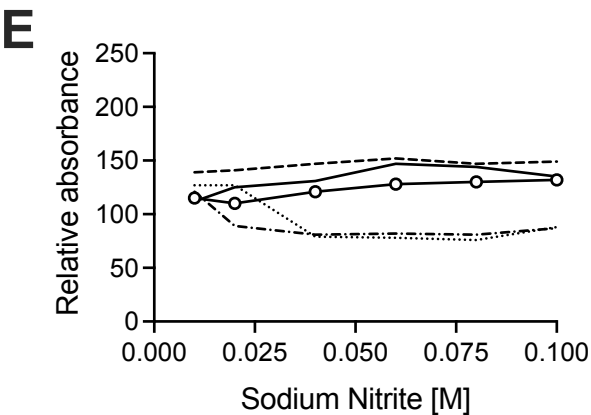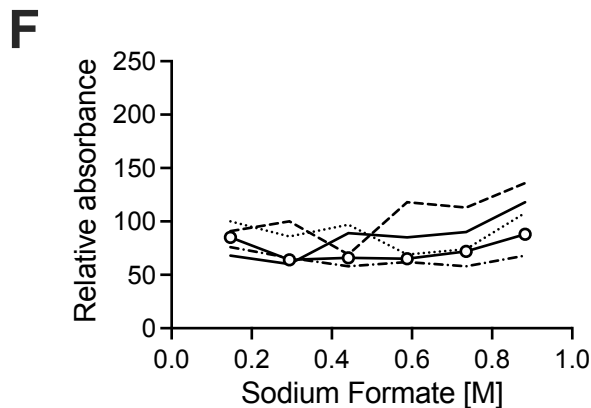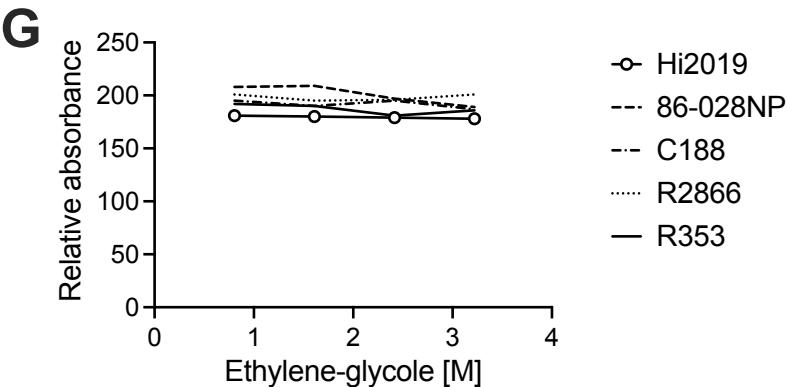

Supplement: S1 Fig — Growth of NTHi strains in the presence of osmotic stressors, phenotypic microarrays (PM09-PM10 plates) A: sodium chloride, B: potassium chloride, C: sodium phosphate, D: ammonium sulfate, E: sodium nitrite, F: sodium formate, G: ethylene glycol. (PDF) [file ppat.1010209.s001.pdf]

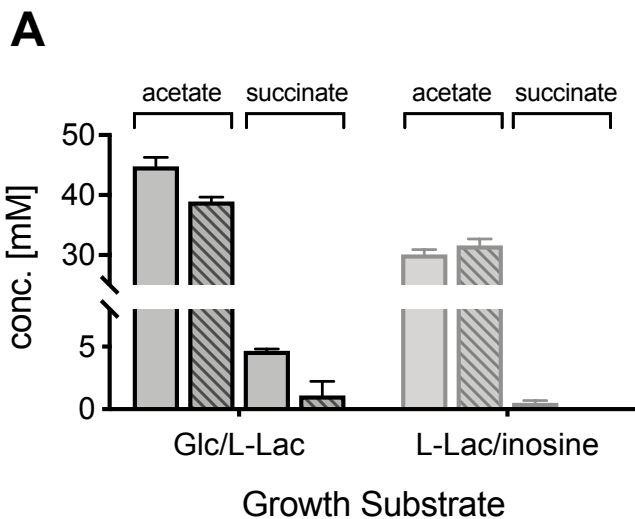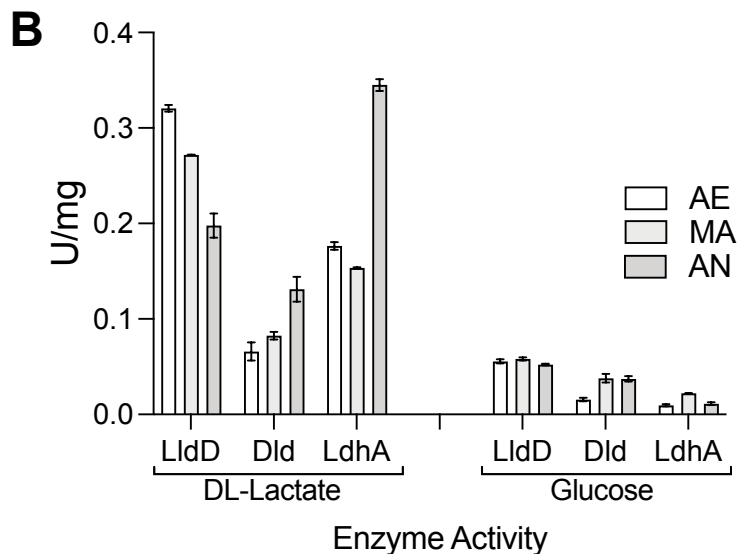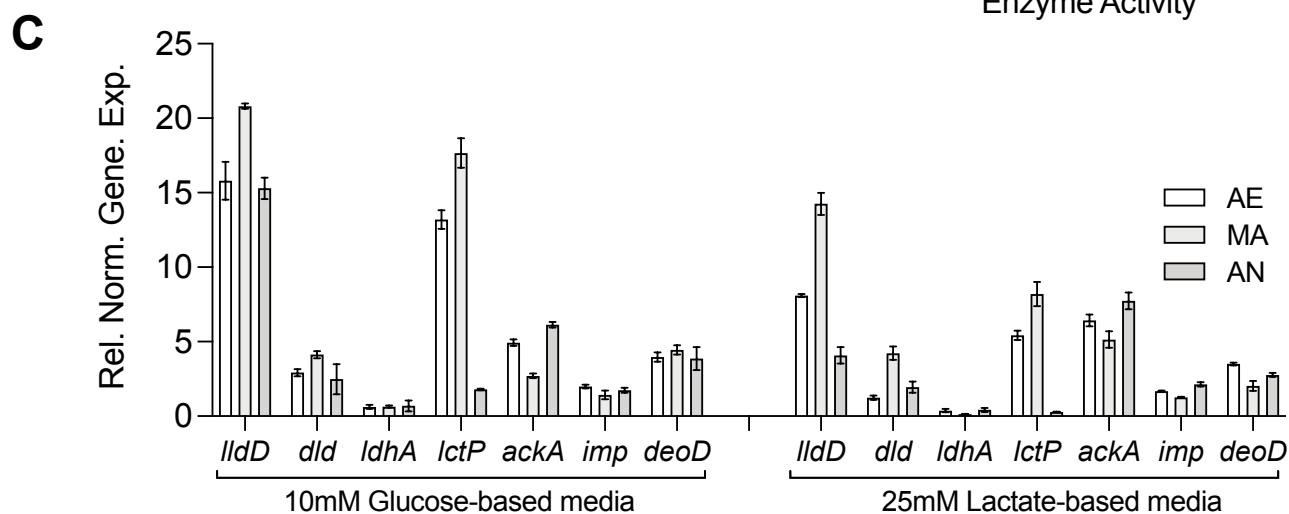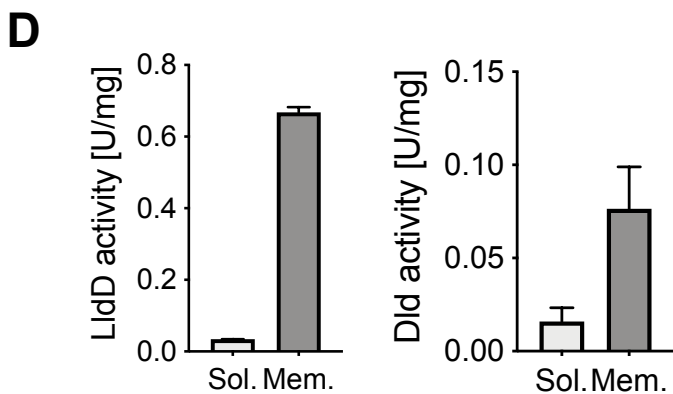

Supplement: S2 Fig — A: Metabolites produced by strains Hi2019 (solid colour) and R2866 (diagonal stripes) during growth on CDM containing glucose & lactate (Glc/L-Lac– 10 mM glucose, 4 mM L-lactate) or lactate & inosine (L-Lac/inosine– 4 mM L-lactate, 7.5 mM inosine). Metabolites were analysed by HPLC. Formate was only detected in samples of Hi2019 following growth with glucose as te carbon source at a concentration of 0.8 ± 0.1 mM and is not shown in the graph as a result. B: Lactate dehydrogenase activities in Hi2019WT following growth on CDM with lactate or glucose as the carbon source. Assays were conducted following growth under aerobic, microaerobic and anaerobic conditions C: gene expression of genes involved in lactate and inosine metabolism in Hi2019 following growth on CDM containing either glucose and L-lactate as the carbon source. D: Distribution of Dld and LldD activity in Hi2019 cell membranes (Mem.) and soluble (Sol.) cell extracts. (PDF) [file ppat.1010209.s002.pdf]

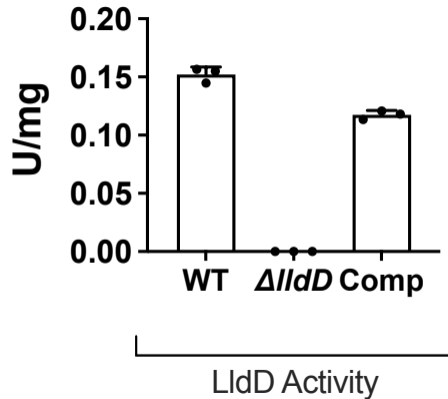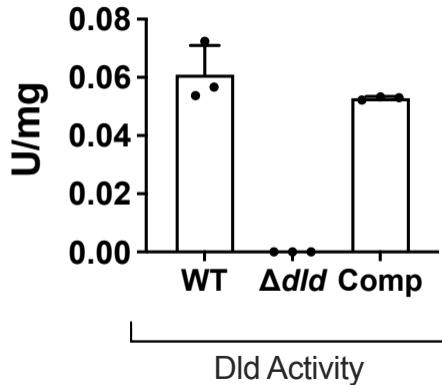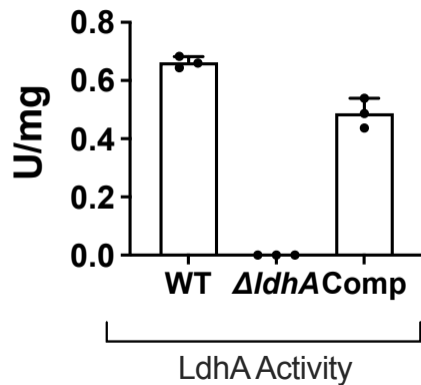

Supplement: S3 Fig — Each assay was conducted using the Hi2019WT strain, the LDH-mutant strain and the complemented strain following growth on CDM with glucose, as some strains (DlldD) were unable to grow on lactate containing media. (PDF) [file ppat.1010209.s003.pdf]

A

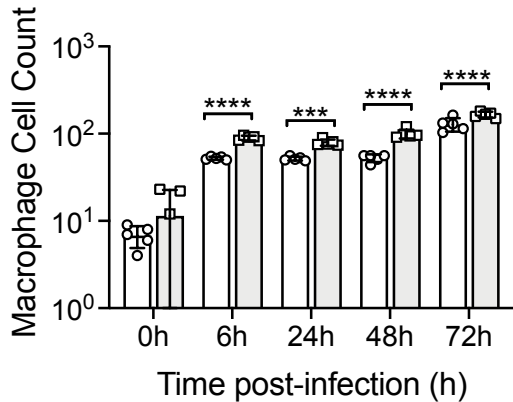

B

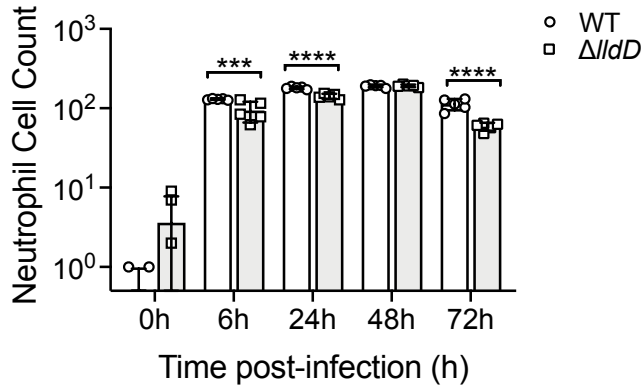

Supplement: S4 Fig — Immune cells were detected using a Giemsa stain A: macrophage cell counts, B: neutrophil cell counts. Statistical analyses used 2-way ANOVA (Sidak post-hoc test), **** p<0.001; *** p<0.001. a p<0.05 was considered statistically significant. (PDF) [file ppat.1010209.s004.pdf]

A

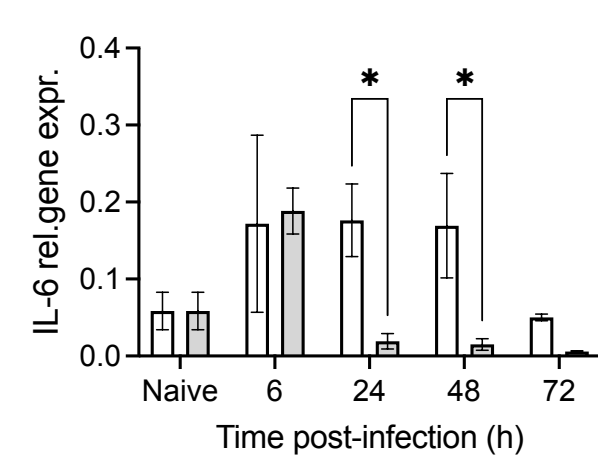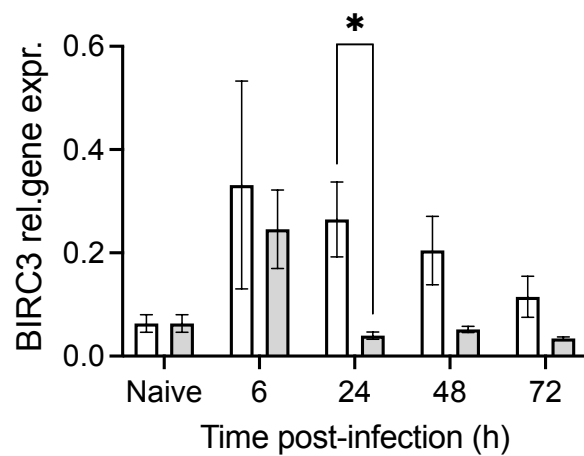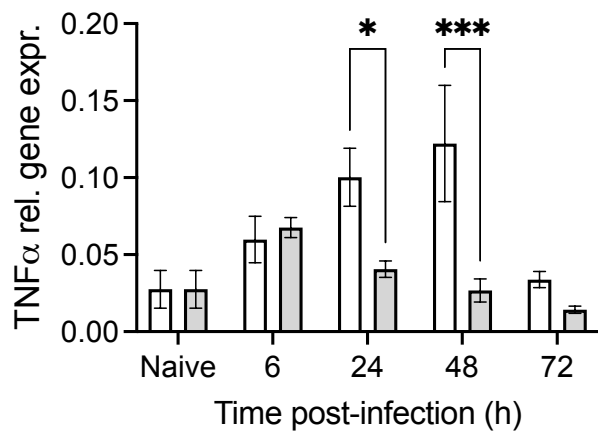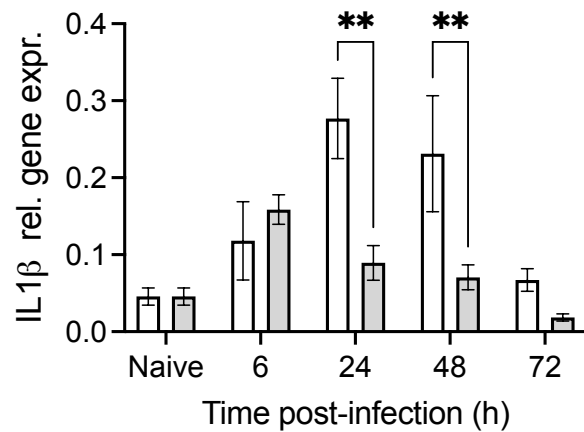

B

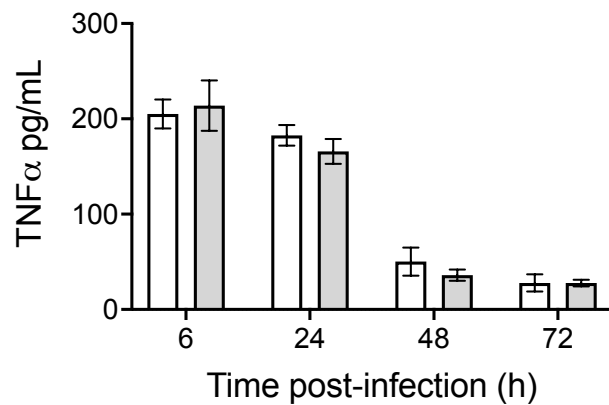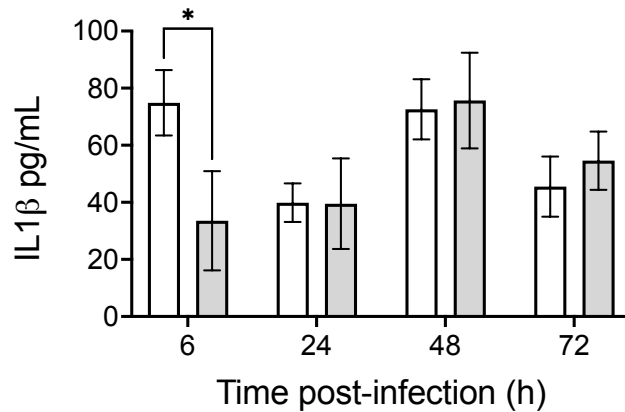

Supplement: S5 Fig — Lung associated immune responses in mice infected with H. influenzae wildtype (white) and lldD mutant (grey) strains. Panel A: Expression of genes encoding key cytokines (IL-6, TNFa, IL-1b) and innate immunity signalling molecular (BIRC3) in mouse lung tissue during infection. Gene expression levels shown are for 3 mice per timepoint and are expressed relative to ACTB. Panel B: Cytokine levels in BALF from infected mice. Levels of TNFa and IL-1b shown are averages of 5 mice per timepoint. Statistical testing used 2-WAY ANOVA (Fisher’s LSD post hoc test), *—p<0.05, **—p<0.01, ***—p<0.0001. (PDF) [file ppat.1010209.s005.pdf]
