# Supplementary material for: Access to highly specialized growth substrates and production of epithelial immunomodulatory metabolites determine survival of Haemophilus influenzae in human airway epithelial cells
Source: PLoS Pathog. 2022 Jan 27;18(1):e1010209. doi: 10.1371/journal.ppat.1010209 (PMC8794153; doi:10.1371/journal.ppat.1010209)
Supplement: S2 Table — Metabolites in brackets have extensive overlap with other NMR signals. MEM–Minimal Essential Medium. (PDF) [file ppat.1010209.s007.pdf]

compound	Concentration (mM)				
		16HBE14		16HBE14 + Hi2019	
	MEM Medium components	4h	24h	4h	24h
**major components**					
Glucose	5.556	5.080±0.076	3.552±0.350	3.995±0.149	1.338±0.201
Lactate	-	1.596±0.225	3.356±0.609	0.528±0.112	1.138±0.066
Ethanol	-	1.733±0.250	3.521±0.562	3.504±0.486	3.084±0.143
Alanine	-	0.180±0.031	0.590±0.143	0.363±0.095	1.772±0.083
Acetate	-	0.109±0.006	0.107±0.008	1.162±0.069	7.253±0.196
Succinate	-	0.063±0.004	0.063±0.012	0.101±0.008	0.362±0.073
Formate	-	0.043±0.001	0.048±0.003	0.058±0.006	0.119±0.072
pyruvate	-	0.032±0.006	0.060±0.010	0.523±0.039	0.032±0.013
**other compounds detected**					
Arginine	0.597	0.340±0.055	0.358±0.040	0.352±0.021	0.406±0.029
(Cystine)	0.100	0.095±0.012	0.126±0.052	0.186±0.009	0.153±0.084
Glutamate	-	0.062±0.023	0.069±0.024	0.033±0.007	0.092±0.038
Glutamine	-	0.146±0.021	0.414±0.421	0.217±0.010	0.939±0.683
(Histidine)	0.200	0.182±0.010	0.089±0.074	0.160±0.006	0.143±0.066
Isoleucine	0.397	0.270±0.088	0.300±0.060	0.288±0.034	0.332±0.018
Leucine	0.397	0.316±0.030	0.275±0.047	0.291±0.043	0.331±0.014
(Lysine)	0.399	0.212±0.009	0.205±0.044	0.219±0.015	0.225±0.039
Methionine	0.101	0.081±0.004	0.075±0.013	0.087±0.005	0.077±0.018
Phenylalanine	0.194	0.156±0.003	0.146±0.027	0.147±0.009	0.146±0.009
Threonine	0.403	0.170±0.014	0.168±0.029	0.171±0.006	0.186±0.005
Tryptophan	0.049	0.031±0.002	0.031±0.007	0.030±0.004	0.010±0.001
Tyrosine	0.199	0.160±0.029	0.134±0.037	0.166±0.028	0.126±0.020
Valine	0.393	0.285±0.019	0.263±0.042	0.281±0.025	0.332±0.017
Choline Chloride	0.007	0.010±0.003	0.016±0.007	0.018±0.003	0.017±0.004
Niacinamide	0.008	0.006±0.000	0.005±0.076	0.006±0.001	0.003±0.003
Citrate	-	0.020±0.000	0.018±0.002	0.000±0.000	0.000±0.000
Glycerol	-	3.410±4.911	0.667±0.206	6.556±5.004	2.083±2.407
Creatine	-	0.015±0.004	0.015±0.002	0.017±0.002	0.069±0.092
Creatine phosphate	-	0.019±0.001	0.013±0.004	0.009±0.000	0.016±0.006
Creatinine	-	0.014±0.005	0.004±0.000	0.023±0.001	0.015±0.009
Hypoxanthine	-	0.007±0.002	0.004±0.002	0.004±0.001	0.001±0.000
Inosine	-	0.002±0.001	0.002±0.000	0.001±0.000	0.001±0.000
