# Supplementary material for: Access to highly specialized growth substrates and production of epithelial immunomodulatory metabolites determine survival of Haemophilus influenzae in human airway epithelial cells
Source: PLoS Pathog. 2022 Jan 27;18(1):e1010209. doi: 10.1371/journal.ppat.1010209 (PMC8794153; doi:10.1371/journal.ppat.1010209)
Supplement: S3 Table — None of the strains was able to grow with D-lactate as the substrate. Shading: highlights conditions with altered growth rates compared to the WT strain. (PDF) [file ppat.1010209.s008.pdf]

**Hi strains**		**WT**	**Δ*lldD***	**Δ*dld***	**Δ*ldhA***
**Glc**	AE	0.507 ± 0.017	0.486 ± 0.017	0.541 ± 0.024	0.520 ± 0.030
	MA	0.426 ± 0.003	0.349 ± 0.011	0.385 ± 0.009	0.433 ± 0.009
	AN	0.321 ± 0.026	0.204 ± 0.075	0.131 ± 0.022	0.308 ± 0.047
**DL-lac.**	AE	0.584 ± 0.040	0±0	0.555 ± 0.009	0.596 ± 0.056
	MA	0.583 ± 0.039	0±0	0.576 ± 0.009	0.600 ± 0.041
	AN	0.193 ± 0.008	0±0	0.274 ± 0.009	0.248 ± 0.014
**L-lac.**	AE	0.531 ± 0.002	0±0	0.395 ± 0.078	0.510 ± 0.050
	MA	0.374 ± 0.059	0±0	0.406 ± 0.027	0.255 ± 0.027
	AN	0.204 ± 0.024	0±0	0.209 ± 0.015	0.265 ± 0.003
