# Supplementary material for: Access to highly specialized growth substrates and production of epithelial immunomodulatory metabolites determine survival of Haemophilus influenzae in human airway epithelial cells
Source: PLoS Pathog. 2022 Jan 27;18(1):e1010209. doi: 10.1371/journal.ppat.1010209 (PMC8794153; doi:10.1371/journal.ppat.1010209)
Supplement: S5 Table — LldD and Dld were assayed with both MTT and DCPIP as artificial electron acceptors, LdhA with pyruvate/NADH as substrates. No LdhA activity was detected with L- or D-lactate/NAD as substrates. kM and Vmax were determined by direct non-linear fitting of data to the Michaelis-Menten equation, values were determined using three biological replicates, error shown: standard deviation. (PDF) [file ppat.1010209.s010.pdf]

| **Enzyme** | **Substrate** | $\boldsymbol{k}_{\mathbf{Mapp}}$ **(mM)** | $\mathbf{V}_{\mathbf{max\_app}}$ **[U/mg]** |
|---|---|---|---|
| **LldD** | L-lactate | 0.13±0.02 (MTT) | 0.28±0.01 (MTT) |
| | | 0.30±0.03 (DCPIP) | 0.34±0.08 (DCPIP) |
| **Dld** | D-lactate | 9.74±1.17 (MTT) | 0.27±0.01 (MTT) |
| | | 0.82±0.08 (DCPIP) | 0.037±0.001 (DCPIP) |
| **LdhA** | pyruvate | 3.38±0.84 | 1.41±0.08 |
| | NADH | 0.055±0.003 | 1.65±0.04 |
