# Supplementary material for: Access to highly specialized growth substrates and production of epithelial immunomodulatory metabolites determine survival of Haemophilus influenzae in human airway epithelial cells
Source: PLoS Pathog. 2022 Jan 27;18(1):e1010209. doi: 10.1371/journal.ppat.1010209 (PMC8794153; doi:10.1371/journal.ppat.1010209)
Supplement: S6 Table — All substrate concentrations are given in mM. Bold font–reduced endproducts concentrations in the lldD mutant strain. (PDF) [file ppat.1010209.s011.pdf]

	WT	Δ*lldD*	Δ*dld*	Δ*ldhA*
substrates				
Glucose	3.704	0.365	0.829	3.262
Inosine	2.232	1.979	2.149	2.021
Pyruvate	0.000	0.000	0.000	0.000
products				
Lactate	0.088	0.059	0.110	0.081
Acetate	11.997	**9.381**	11.670	11.335
Succinate	12.448	**9.143**	12.215	11.586
Formate	4.780	**2.726**	4.321	4.452
Hypoxanthine	4.275	4.115	4.478	3.987
Fumarate	0.054	0.016	0.039	0.051
