# Supplementary material for: Access to highly specialized growth substrates and production of epithelial immunomodulatory metabolites determine survival of Haemophilus influenzae in human airway epithelial cells
Source: PLoS Pathog. 2022 Jan 27;18(1):e1010209. doi: 10.1371/journal.ppat.1010209 (PMC8794153; doi:10.1371/journal.ppat.1010209)
Supplement: S7 Table — (PDF) [file ppat.1010209.s012.pdf]

**Table S7** – Bacterial strains and plasmids used in this study.

| **Strain** | **Description** | **Reference or Source** |
|---|---|---|
| *Escherichia coli* DH5α | $F^-$ φ80*lac*ZΔM15 Δ(*lac*ZYA-*arg*F)U169 *rec*A1 *end*A1 *hsd*R17($r_K^-$, $m_K^+$) *pho*A *sup*E44 $\lambda^-$ *thi*-1 *gyr*A96 *rel*A1, cloning strain | Life Technologies |
| *Haemophilus influenzae* 2019 (Hi2019 WT) | Clinical isolate from a chronic obstructive pulmonary disease patient.<br>Sequence type 321 | Campagnari, Gupta (1) |
| *Haemophilus influenzae* 86-028NP | Clinical isolate from a patient with otitis media.<br>Sequence type 33 | Harrison, Dyer (2) |
| *Haemophilus influenzae* C188 | Clinical isolate from blood.<br>Sequence type 269 | Kappler, Dhouib (3) |
| *Haemophilus influenzae* R2866 | Clinical isolate from the blood of a patient with otitis media.<br>Sequence type 99 | Nizet, Colina (4) |
| *Haemophilus influenzae* R535 | Clinical isolate from sputum.<br>Sequence type: unknown | Kappler, Dhouib (3) |
| Hi2019Δ*lldD* | Hi2019 WT with *lldD* gene disrupted by the insertion of a kanamycin antibiotic resistance cassette<br>(*lldD::*kan) | This study |
| Hi2019Δ*dld* | Hi2019 WT with *dld* gene disrupted by the insertion of a kanamycin antibiotic resistance cassette<br>(*dld::*kan) | This study |
| Hi2019Δ*ldhA* | Hi2019 WT with *ldhA* gene disrupted by the insertion of a kanamycin antibiotic resistance cassette<br>(*ldhA::*kan) | This study |
| Hi2019Δ$lldD^{lldD_comp}$ | Hi2019Δ*lldD* containing a functional copy of *lldD*<br>(Δ*lldD::lldD)* | This study |
| Hi2019Δ$dld^{dld_comp}$ | Hi2019Δ*dld* containing a functional copy of *dld*<br>(Δ*dld::dld)* | This study |
| Hi2019Δ$ldhA^{ldhA_comp}$ | Hi2019Δ*ldhA* containing a functional copy of *ldhA*<br>(Δ*ldhA::ldhA)* | This study |
| Hi2019Δ*IMPDH* | Hi2019 WT with *guaB* gene disrupted by the insertion of a kanamycin antibiotic resistance cassette<br>(*guaB::*kan) | This study |
| | | |
| **Plasmid** | **Description** | **Reference or Source** |

| | | |
|---|---|---|
| pUC4K | Cloning vector used to isolate the kanamycin resistance cassette. Vector also contains ampicillin resistance cassette. | [5] |
| pGEM-T Easy | Cloning vector | Promega |
| pGEM-Hi-*lldD* | pGEM-T Easy derivative containing a 1000bp DNA fragment carrying the *lldD* gene and flanking regions | This study |
| pGEM-Hi-*lldD*::kan | pGEM-Hi-*lldD* with the *lldD* gene disrupted by a kanamycin antibiotic resistance cassette | This study |
| pBluescript II SK+ | Cloning vector | Stratagene |
| pBlue-Hi-*dld* | pBluescript derivative containing a 3098bp DNA fragment carrying the *dld* gene and flanking regions | This study |
| pBlue-Hi-*dld*::kan | pBlue-Hi-*dld* with *dld* gene disrupted by a kanamycin antibiotic resistance cassette | This study |
| pBlue-Hi-*ldhA* | pBluescript derivative containing a 2429bp DNA fragment carrying the *ldhA* gene and flanking regions | This study |
| pBlue-Hi*l-dhA*::kan | pBlue-Hi-*ldhA* with *ldhA* gene disrupted by a kanamycin antibiotic resistance cassette | This study |
| pBlue-Hi-*IMPDH* | pBluescript derivative containing a 1880bp DNA fragment carrying the *guaB* gene and flanking regions | This study |
| pBlue-Hi-*IMPD*H::kan | pBlue-Hi*IMPDH* with *guaB* gene disrupted by a kanamycin antibiotic resistance cassette | This study |
| p601.1-sp2 | Cloning vector containing Hi2019 601.1 pseudogene fragments flanking the insertion region and a spectinomycin resistance cassette | Johnston, Zaleski (6) |
| p601-Hi-*lldD* | p601.1-sp2 derivative containing a 1200bp DNA fragment carrying a functional copy of the *lldD* gene region | This study |
| p601-Hi-*dld* | p601.1-sp2 derivative containing a 2549bp DNA fragment carrying a functional copy of the *dld* gene region | This study |
| p601-Hi-*ldhA* | p601.1-sp2 derivative containing a 2208bp DNA fragment carrying a functional copy of the *ldhA* gene region | This study |

References


1. Campagnari AA, Gupta MR, Dudas KC, Murphy TF, Apicella MA. Antigenic diversity of lipooligosaccharides of nontypable *Haemophilus influenzae*. Infect Immun. 1987;55(4):882-7. PubMed PMID: 3549563; PubMed Central PMCID: PMCPMC260433.

2. Harrison A, Dyer DW, Gillaspy A, Ray WC, Mungur R, Carson MB, et al. Genomic sequence of an otitis media isolate of nontypeable *Haemophilus influenzae*: comparative study with *H. influenzae* serotype d, strain KW20. J Bacteriol. 2005;187(13):4627-36. doi: 10.1128/JB.187.13.4627-4636.2005. PubMed PMID: 15968074.

3. Kappler U, Dhouib R, Nair RP, McEwan AG. Draft genome sequences of three nontypeable strains of *Haemophilus influenzae*, C188, R535, and 1200, isolated from different types of disease. Genome Announc. 2017;5(12):e00035-17. doi: 10.1128/genomeA.00035-17. PubMed PMID: 28336588.

4. Nizet V, Colina KF, Almquist JR, Rubens CE, Smith AL. A virulent nonencapsulated *Haemophilus influenzae*. J Infect Dis. 1996;173(1):180-6. doi: 10.1093/infdis/173.1.180.

5. Vieira J, Messing J. The pUC plasmids, an M13mp7-derived system for insertion mutagenesis and sequencing with synthetic universal primers. Gene. 1982;19(3):259-68. doi: https://doi.org/10.1016/0378-1119(82)90015-4.

6. Johnston JW, Zaleski A, Allen S, Mootz JM, Armbruster D, Gibson BW, et al. Regulation of sialic acid transport and catabolism in *Haemophilus influenzae*. Mol Microbiol. 2007;66(1):26-39. Epub 2007/09/21. doi: 10.1111/j.1365-2958.2007.05890.x. PubMed PMID: 17880422
