# Supplementary material for: Access to highly specialized growth substrates and production of epithelial immunomodulatory metabolites determine survival of Haemophilus influenzae in human airway epithelial cells
Source: PLoS Pathog. 2022 Jan 27;18(1):e1010209. doi: 10.1371/journal.ppat.1010209 (PMC8794153; doi:10.1371/journal.ppat.1010209)
Supplement: S8 Table — Table adapted from Biolog Inc, Hayward, CA, USA (https://www.biolog.com/products-portfolio-overview/phenotype-microarrays-for-microbial-cells/). (PDF) [file ppat.1010209.s013.pdf]

Composition and Preparation of 12x PM Additive Solutions							
**Ingredients**	**Stock Conc.**	**Conc. in PM Additive (12x)**	**Vol. of solutions required for each plate type**				
			**PM1,2**	**PM3,6,7,8**	**PM4**	**PM5**	**PM9+**
Tricarballylic acid (pH7.1)	800mM	240mM	-	6ml	6ml	6ml	-
$MgCl_2$, 6H_2O<br>$CaCl_2$, 2H_2O	240mM<br>120mM	24mM<br>12mM	2ml	2ml	2ml	2ml	2ml
L-arginine, HCl<br>L-glutamic acid	3mM<br>6mM	300µM<br>600µM	2ml	-	2ml	-	-
b-NAD, Na<br>hypoxanthine<br>uracil<br>L-cystine (pH8.5)	0.2mM<br>1mM<br>1mM<br>1mM	60µM<br>300µM<br>300µM<br>300µM	6ml	6ml	-	-	-
Yeast extract	0.6%	0.06%	2ml	2ml	2ml	-	2ml
Tween 80	0.6%	0.06%	2ml	2ml	2ml	-	2ml
D-glucose<br>Pyruvate, Na	300mM<br>600mM	30mM<br>60mM	-	2ml	2ml	2ml	2ml
Sterile water	N/A	0 – 60ml	6ml	-	4ml	10ml	12ml
Total volume			20ml	20ml	20ml	20ml	20ml
**Recipe for 1x PM Inoculating Fluids from Stock Solutions**							
**PM Stock Solution**			**PM1,2**	**PM3,6,7,8**	**PM4**	**PM5**	**PM9+**
IF-0a GN/GP (1.2x)			20.0ml	40ml	10ml	10ml	-
IF-10b GN/GP (1.2x)			-	-	-	-	110ml
Dye Mix G (100x)			0.24ml	0.48ml	0.12ml	0.12ml	1.32ml
PM additive (12x)			2.0ml	4.0ml	1.0ml	1.0ml	11.0ml
Hi cells (turbidity)			65%	65%	65%	65%	65%
Total volume (up to)			24.0ml	48.0ml	12.0ml	12.0ml	132.0ml
