# Supplementary material for: Access to highly specialized growth substrates and production of epithelial immunomodulatory metabolites determine survival of Haemophilus influenzae in human airway epithelial cells
Source: PLoS Pathog. 2022 Jan 27;18(1):e1010209. doi: 10.1371/journal.ppat.1010209 (PMC8794153; doi:10.1371/journal.ppat.1010209)
Supplement: S9 Table — (PDF) [file ppat.1010209.s014.pdf]

Table S9 – Oligonucleotide primers used in this study

Gene Target	Primer Name	Primer Sequence (5' – '3)
**Hi mutant construction primers**		
KO-*lldD*	Hi2019*lldD*-KO-F	AAAACGAAGGTCCCACACTTTAATC
	Hi2019*lldD*-KO-R	AAAACACATTTTCCCGAAGAATCTC
Comp-*lldD*	Hi2019*lldD*-Comp-F	AAAA*CCCGGG* AAACAAGTCAATCAACAAGTGCC
	Hi2019*lldD*-Comp-R	AAAA*CCCGGG* GCTTTACATCTATGGGGATTTGA
Upstream region of *ldhA*	Hi2019*ldhA*-upF-PstI	AAAA*CTGCAG* GATACTACGAACACCAAATTGTGC
	Hi2019*ldhA*-upR-BamHI	AAAA*GGATCC* CCATCGGTTTGATGATGACTC
Downstream region of *ldhA*	Hi2019*ldhA*-downF-BamHI	AAAA*GGATCC* TATGTTCCGCCACCGCTTCAGG
	Hi2019*ldhA*-downR-SacII	AAAA*CCGCGG* GCTCAAGCAGGCAATCGCAGTG
Comp-*ldhA*	Hi2019*ldhA*C-F-XmaI	AAAA*CCCGGG* CCAAAAACTAAGACGGCAAAA
	Hi2019*ldhA*C-R-XmaI	AAAA*CCCGGG* CACTTTTTCCTTTGTGCCAAA
Upstream region of *dld*	Hi2019*dld*-upF-PstI	AAAA*CTGCAG* ATGCGGACTGAATCATCTATGA
	Hi2019*dld*-upR-BglII	AAAA*AGATCT* AGGCTCCCCTGCTCGTTTTA
Downstream region of *dld*	Hi2019*dld*-downF-BglII	AAAA*AGATCT* TCATCAACTTGACGAACATAGT
	Hi2019*dld*-downR-SacI	AAAA*GAGCTC* CTAGAACCTACACTCAATCAC
Comp-*dld*	Hi2019*dld*C-F-XmaI	AAAA*CCCGGG* TGCGGTAAACATCAGATAATTTGC
	Hi2019*dld*C-R-XmaI	AAAA*CCCGGG* CGGCTTGTTTCATCATTTGGTTAA
Upstream region of *IMPDH*	Hi2019*IMPDH*-upF1-SacI	AAAA*GAGCTC* AATCTTGTCCATCAACGAGAATATC
	Hi2019*IMPDH*-downR1-BglII	AAAA*AGATCT* GGAAGTCTTTAACGGTAATCATACC
Downstream region of *IMPDH*	Hi2019*IMPDH*-upF2-BglII	AAA*AAGATCT* AAAAAGCAGAACAAAAACCAAATG
	Hi2019*IMPDH*-downR2-SacII	AAAA*CCGCGG* GATCAGGATTTTGTGATTGTGGATA
Kan^r cassette with flanking regions	pUC4K-PCR-F	GTTGGGTAACGCCAGGGTTTTCC
	pUC4K-PCR-R	TCCGGCTCGTATGTTGTGTGGAA
Kanamycin resistance cassette	pUC4K-F	GGAAAGCCACGTTGTGTCTC
	pUC4K-R	CTGAGGTCTGCCTCGTGAAG
**qPCR primers**		
Human IL-6	HS-QP-IL6_F	TGCCCCAGTACCCCCAGGAGAAGAT

	HS-QP-IL6_R	AGGGCTGAGATGCCGTCGAGGATGT
Human IL-8	HS-QP-IL8_F	GGTGCAGTTTTGCCAAGGAGTGCTA
	HS-QP-IL8_R	GCGCAGTGTGGTCCACTCTCAATCA
Human IL-1β	HS-QP-IL1Beta_F2	GCTCGCCAGTGAAATGATGGCTT
	HS-QP-IL1Beta_R2	CCATCCAGAGGGCAGAGGTCCA
Human ACTB	HS-QP-ACTB-F	TGCTATCCCTGTACGCCTCTGGC
	HS-QP-ACTB-R	CCAGACGCAGGATGGCATGG
Hi2019 *lctP*	Hi-QP-lctP_F	TACCAAAACCACTTGCCCCCTCGAT
	Hi-QP-lctP_R	CAGGCGCAACAAATATTATGCGAAA
Hi2019 *ldhA*	HI-RT-ldhAF	CTGGCGTTGCTGGGCAATGA
	HI-RT-ldhAR	TCTTGGCGTACGATCCTTTCA
Hi2019 *lldD*	HI-RT-lldDF	ATCATACGCCCAGAATGGGTG
	HI-RT-lldDR	ACGGTCGATATGCCTACTCCA
Hi2019 *dld*	HI-RT-dldF	AGCGGAAAGGTGTCTAAACGA
	HI-RT-dldR	TCGTCAAGTTGATGAAGGCTC
Hi2019 *guaB*	HI-QP-guaBF	CAGGCGCAAGTGCTGTAAAAGTAGG
	HI-QP-guaBR	CTTTTAATGCCGCCGCTGCA
Hi2019 *deoD*	HI-QP-deoDF	TCACGTCCATACGAACAGTGCCAC
	HI-QP-deoDR	TTATGGGGCACGGAATGGGG
Hi2019 *gyrA*	HI-Qp-gyrAF	GCGTGCATTACCTGACGTTCGAG
	HI-Qp-gyrAR	CCCACAACACGCGCTGATTTTAC
Mouse ACTB	Mm-QP-ACTB-F	CTGCGTCTGGACCTGGC
	Mm-QP-ACTB-R	CTTCTCTTTGATGTCACGCACGAT
Mouse BIRC3	Mm-QP-BIRC3-F	CTGTGTCAGAAAGGAGTCTGGCT
	Mm-QP-BIRC3-R	CCATGGGACTGTCCCCTTG
Mouse IL-1β	Mm-QP-IL1beta-F	GCTTCAAATCTCGCAGCAGC
	Mm-QP-IL1beta-R	TCCTCATCCTGGAAGGTCCAC
Mouse TNFα	Mm-QP-TNFalpha-F	TGAGCACTGAAAGCATGATCCG
	Mm-QP-TNFalpha-R	CGATCAGGAAGGAGAAGAGGCTG
Mouse IL-6	Mm-QP-IL6-F	GACTTCCATCCAGTTGCCTT
	Mm-QP-IL6-R	GGTATAGACAGGTCTGTTGG
